# Importance of Mineral Nutrition for Mitigating Aluminum Toxicity in Plants on Acidic Soils: Current Status and Opportunities

**DOI:** 10.3390/ijms19103073

**Published:** 2018-10-08

**Authors:** Md. Atikur Rahman, Sang-Hoon Lee, Hee Chung Ji, Ahmad Humayan Kabir, Chris Stephen Jones, Ki-Won Lee

**Affiliations:** 1 Molecular Breeding Laboratory, Grassland and Forages Division, National Institute of Animal Science, Rural Development Administration, Cheonan 31000, Korea; atikgnu@gmail.com (M.A.R.); sanghoon@korea.kr (S.-H.L.); cornhc@korea.kr (H.C.J.); 2 Molecular Plant Physiology Laboratory, Department of Botany, University of Rajshahi, Rajshahi 6205, Bangladesh; ahmad.kabir@ru.ac.bd; 3 Feed and Forage Biosciences, International Livestock Research Institute, P.O. Box 5689, Addis Ababa, Ethiopia; c.s.jones@cgiar.org

**Keywords:** mineral nutrient, aluminum, toxicity, alleviation, plant, acidic soil

## Abstract

Aluminum (Al) toxicity is one of the major limitations that inhibit plant growth and development in acidic soils. In acidic soils (pH < 5.0), phototoxic-aluminum (Al^3+^) rapidly inhibits root growth, and subsequently affects water and nutrient uptake in plants. This review updates the existing knowledge concerning the role of mineral nutrition for alleviating Al toxicity in plants to acid soils. Here, we explored phosphorus (P) is more beneficial in plants under P-deficient, and Al toxic conditions. Exogenous P addition increased root respiration, plant growth, chlorophyll content, and dry matter yield. Calcium (Ca) amendment (liming) is effective for correcting soil acidity, and for alleviating Al toxicity. Magnesium (Mg) is able to prevent Al migration through the cytosolic plasma membrane in root tips. Sulfur (S) is recognized as a versatile element that alleviates several metals toxicity including Al. Moreover, silicon (Si), and other components such as industrial byproducts, hormones, organic acids, polyamines, biofertilizers, and biochars played promising roles for mitigating Al toxicity in plants. Furthermore, this review provides a comprehensive understanding of several new methods and low-cost effective strategies relevant to the exogenous application of mineral nutrition on Al toxicity mitigation. This information would be effective for further improvement of crop plants in acid soils.

## 1. Introduction

Aluminum (Al) toxicity represents a serious limitation to plant production in acid soils worldwide, as approximately 40–50% of the world’s total potential arable land consists of acidic soils [1] Acid soils (pH 5.5 or lower) are globally distributed and comprise approximately 30% of the total area of the earth [2]. Hence, soil acidification is a natural process which occurs mostly in tropical and subtropical regions. Several natural and/or anthropogenic inputs are responsible for accelerating soil acidification [3]. The important causes of soil acidification on agricultural land are acidic precipitation (H^+^ ions in precipitation), input of acidifying gasses or particles (i.e., SO_2_; NO_3_), contribution of nitric and hydrochloric acids (i.e., HNO_3_; HCl) from the atmosphere, application of elemental sulfur (S), ammonium-based fertilizer (NH_4_^+^), nutrient uptake by leguminous crops, and mineralization of organic matter [4,5,6]. 

Impacts of soil acidification have been critically recorded on agricultural soil as well as pH level. The replacement of exchangeable base cations such as calcium (Ca^2+^), magnesium (Mg^2+^) and potassium (K^+^) by H^+^ and Al^3+^, and the dissolution of Al-bearing and Mn minerals, and the dissolution of Fe-bearing minerals are the most significant consequences of soil acidifications. These three processes buffer the soil pH at approximately 5–6, 4, and 3, respectively [5]. Consequently, metal toxicity (i.e., Mn, Fe, and Al) and nutrient imbalance (i.e., P) are found to occur in acid soils, wherein Al toxicity is the most significant threat to plant survival in acid soils [3].

Al is the most abundant metal on earth; it is ubiquitously distributed as the third most abundant element in the earth’s crust that comprising 7–8% of its mass after oxygen and silicon [3,6]. However, the specific biological function of Al is still to be disclosed. The presence of Al could be marked easily in all forms of life as it is an integral component of mineral soil. Country specific soil acidity and concentration of Al in soils has been widely distribution worldwide (Table 1). Al in the soil is mainly found incorporated in the form of minerals such as aluminum oxides or harmless aluminosilicates [7]. However, solubilization and speciation of Al depend on the chemical environment and the pH of the soil solution [8]. In acid soil at low pH (4.3), Al is solubilized into [Al(H_2_O)_6_]^3+^ that usually referred as to Al^3+^. Al also formulates other species such as Al(OH)^2+^, Al(OH)_2_^+^, Al(OH)_3_^−^, and Al(OH)_4_^−^, wherein Al^3+^ is considered as the most toxic form that has a huge impact on plant growth and development [6,7,9]. One of the major consequences and the most obvious symptom of Al toxicity is the root growth inhibition in plants [10]. Excessive Al inhibits roots cell division–elongation, root hair formation, and enhances the development of swollen roots apices [11]. Concurrently, toxic Al inhibits the uptake of water and nutrients by plants [3]. Several reports have provided indications that toxic Al^3+^ alters nutrient levels such as N, K, Ca, Mg, and P, and reduces the photosynthetic rate (*P_N_*), stomatal conductance (gs), and leaf transpiration (*E*) rate in plants [7,12,13]. Surprisingly, the initial response to Al toxicity marked to be induced within a few minutes even at micro-molar (µM) concentrations of toxic Al in plant cells [14]. The phytotoxic Al leads to generate excess reactive oxygen species (ROS) such as H_2_O_2_ and O_2_, as those ROS were detected in the root tips of *Glycine max* [15], the leaves of *Oryza sativa* [16], and cells of *Nicotiana tabacum* [17]. Other phytotoxic effects of Al have been described in different cellular organelles; such as toxic Al-induced disruption of free cytosolic Ca^2+^ [18], callose deposition at the plasmodesmata [19], and respiration inhibition in mitochondria [20]. 

Several studies have shown the impact of Al toxicity on crop plants based on their sensitivity threshold to acid soils; wherein most of them mainly focused on roots and their growth [21,22,23]. Other issues are included like Al protection, tolerance, and/or resistance mechanisms [13,24,25,26], Al effects on plant metabolism [7], Al speciation and detoxification in plants [9], the role of mitochondria in the Al response [27], the link between Al chemistry and biology [8], inhibition of auxins synthesis, and transportation inhibition by Al toxicity [23], have been extensively studied in plants. Recently, it has been elucidated both toxic and beneficial impacts of Al in plants [3], also clarified the accumulation, localization, and impacts of Al on various levels of the plant organs [6], and identified the Al-induced genes in the root apex of buckwheat [26]. Molecular and physiological mechanisms related to Al resistance and/or tolerance are extremely complex phenomena. As a part of natural selection, plants have evolved some specific mechanism to cope with Al toxicity. Exclusion was extensively mentioned as it the key approach to detoxifying Al toxicity in plants [25]. Exudation chelating ligands, speciation of pH barriers at the root apoplasm or at the rhizosphere, immobilization of cell walls, selective permeability of the plasma membrane (PM), and Al efflux have been widely suggested as the mechanisms of Al-exclusion [13,21,24,25]. However, these resistance and tolerance mechanisms in plants are not mutually exclusive; rice (*Oriza sativa*) and buckwheat (*Fagopyrum esculentum*) for example benefit from both mechanisms [28]. Al is a non-biodegradable metal that widely distributed in soil environmentglobally [29]. Al is able to be transmitted through the food cycle. The impact of Al toxicity has been manifested not only in plants but also in animals and humans; thus, Al represents a critical threat to the whole system. For example, Al toxicity was reported to be involved in poor quality forage and fodder production, consequently it negatively impacts on grazing animals, cow milk and overall livestock production [30]. Chaneysuggested that 1000 mg Al·kg^−1^ should be the maximum in animal diet, although no results have been given for the time course analysis [31]. At the beginning of the 21st century, soil acidity along with Al-toxicity related damage in crops resulted in huge economic losses of more than 600 million USD in the agricultural sector in Australia [32]. Al is also recognized as a risk factor for human health. For instance, tea leaves appear to accumulate substantial amounts of Al [33], and one-third of total Al is able to be transferred easily during the tea leaf infusion process, this Al can cause potential health problems for humans [34]. Although, a trace amount of Al is reported to be available for absorption across the gastro-intestinal tract [35], tea-drinkers should be warned about this health risk. In contrast, high levels of Al can induce chronic renal failure in humans [36]. Based on several investigations, it has been hypothesized that high levels of Al in the human body are related to several diseases including osteomalacia fractures, encephalopathy, Parkinsonism dementia, and Alzheimer’s disease [37,38,39]. 

Several conventional strategies for farmers have been proposed to ameliorate Al toxicity and/or decrease Al-accumulation through liming, P fertilizer, and the production of low Al-accumulating cultivars through genetic manipulation [13]. In addition, developing a variety with new traits may take 5–10 years, and the entire process involves in a considerable amount of time and expense [40]. Moreover, continuous application of P fertilizer and lime in soil is not only expensive but also environmentally risky [41]. Therefore, low-cost effective and environmentally friendly approaches are in high demand. In this regard, the application of mineral nutrition would be a suitable strategy for minimizing Al toxicity in plants to acid soils. 

Many crop plants have a range of susceptibility to acidic soils, and overall their performance is highly influenced by Al toxicity [42]. Therefore, Al toxicity has emerged as the major limitation to agronomic performance in acidic soils. We updated here the information of several literature reviews provide the evidence on the exogenous application of mineral nutrients mitigating Al toxicity in plants. Consequently, the application of mineral nutrients to mitigate Al toxicity in plants exposed to acid soils is now the most recent and important research topic in this field.

The application of mineral nutrition provides several benefits such as (i) a cost-effective approach relative to other organic and inorganic amendments, (ii) little or low environmental risk of application, (iii) high availability of nutrients, (iv) few skills are needed to sustain crop yield, and (v) crop plants can easily take up mineral nutrition throughout the entire year. Therefore, more attention has been given to study plant–nutrient–soil interactions as well as to minimize Al toxicity in plants exposed to acid soil by nutritional amendments. The main objectives of this review were to: (a) present a comprehensive discussion of several factors affecting soil acidification, Al toxicity, and Al accumulation in plants; (b) understated how plant nutrition can be an effective strategy to minimize Al toxicity; (c) analyze how plants develop suitable strategies to enhance Al toxicity tolerance in the presence of mineral nutrition; and (d) explore the best nutrition management practice for minimizing Al toxicity in plants. This review updates the knowledge concerning the influence and distribution of Al toxicity in crop plants grown in acidic conditions, factors affecting Al toxicity and nutritional imbalance and homeostasis, and overall mechanisms related to the efficiency of different mineral nutrition strategies to mitigate Al toxicity in plants. Captivating materials have been distributed in the current literature pinning down Al toxicity at various levels of plant cells and organisms, the application of mineral nutrition would be an effective strategy to counteract Al toxicity. These updated findings might disclose new avenues for minerals leading to physiological, molecular and agricultural inquiries into Al toxicity that markedly advances our understanding.

## 2. Multiple Forms of Aluminum in the Soil Environment Relevant to Toxicity

Al represents approximately 7–8% of the total solid matter in the earth’s crusts, it ubiquitously distributed as different forms in soil environments [3,6]. Different forms of Al may exist in the soil such as inorganic, soluble, and/or organic forms. Inorganic forms of Al are exchangeable and are primarily bound to silicate clays, hydrous oxides, phosphates, and sulfates [60]. A significant correlation has been found between soil pH and phytotoxicity of Al species. Hence, these multiple forms of Al, their concentrations, speciation, and toxicity in the soil environment depend on pH level and the chemistry of the soil solution [8].

Several soluble forms of Al species such as Al^3+^, Al(OH)^2+^, Al(OH)_2_^+^, and Al(OH)_4_^−^ have been shown to occur when the soil pH drops below 5 [8,61]. In acidic soil (pH < 5), Al is solubilized into [Al(H_2_O)_6_]^3+^, usually referred to as Al^3+^. The solubilization of Al occurs due to the inception of soil acidification which leads to the release of phytotoxic Al^3+^ [6]. This trivalent Al^3+^, which is the most abundant form and very toxic, has the greatest impact on plant growth at pH < 4.3. In contrast, solution pH level increases and reduces the Al^3+^ concentration by repeated deprotonation [8]. At pH > 5–6, mononuclear species are reported to be formed, including Al(OH)^2+^ and Al(OH)_2_^+^, which are toxic to the dicotyledonous plants but not toxic as Al^3+^ is initiated [3]. At pH 7, the formation of gibbsite [Al(OH)_3_], occurs; however, it is non-toxic in nature and relatively insoluble [62]. At alkaline pH (>7), aluminate (Al(OH)_4_^−^) was reported to be formed [63]. Al(OH)_4_^−^ is not always toxic; for example, at a concentration of 25 micromolar it was shown to be non-toxic in red clover whereas in wheat it showed at pH 8–8.9 [64]. Despite the above-mentioned speciation of Al, a highly toxic polynuclear Al species that is identified as “Al_13_”, is reported to be toxic ten-fold higher than Al^3+^ [61]. Surprisingly, the soluble or exchangeable aluminum (Al^3+^) is able to associate with a variety of organic and inorganic ligands. However, organic Al is formed when Al^3+^ binds to several ligands (i.e., SO_4_^2−^, PO_4_^3−^, F^−^ etc.) in the soil solution to generate stable complexes [62]. Exchangeable Al^3+^ cations and assimilable Al can participate in the formation of the above types of complexes. Briefly, the above-mentioned facts suggest that Al speciation, level of Al toxicity, availability of Al in the soil, alteration of pH level, and complexation of ligands are influenced by the chemistry of soil environment. 

## 3. Aluminum Uptake, Accumulation, and Toxicity Responses in Plants

The uptake of Al from the soil by vascular plants is a complex process that is highly influenced by the soil pH, and the chemical environment of the rhizosphere. Due to the lack of conclusive evidence on precise mechanisms regarding Al uptake, some questions about this issue have still to be answered. For example, in which form and process Al are taken up? Which is the most active cellular site for Al uptake? Where is the location of Al-loading into the xylem, and how the process is mediated? Al ions are taken up by plants mostly through the root system, and only to a limited extent do they penetrate the leaves. A body of knowledge indicates that Al is taken up as Al^3+^ by an active process wherein root apices play a vital role in Al toxicity perception and response [9,62]. Though it is still a matter of debate whether Al uptake at the root surface occurs symplastically or apoplastically? Lazof et al. [29] elucidated that a sizable amount of Al efficiently enters the root symplasm, but later it can affect the growth of the membrane on the cytosolic side. Surprisingly, the highest ratio of symplastic Al to total Al has been found at the root apex in buckwheat [22]. Rengel [65] suggested that the entrance pathway of Al^3+^ is the root apoplast, as he found that approximately 30–90% of the total Al that is present in the root apoplasm could be acquired by a plant. Recently, an apoplasmic lesion caused by Al, wherein it is primarily bound to the outer cell wall after immediate exposure has been explored [66].

Based on the Al concentration and organic acid exudation in root systems, several researchers have tried to determine the most active site for Al uptake in plants [9]. The mature elongation zone above the root apex has been suggested as the Al uptake site in buckwheat, as the highest level of oxalic acid exudated from the apex [67]. Klug et al. [22] detected a high proportion of total Al at the root apex, thus, they suggested that the root apex was the most active site of Al uptake in plants. Transporters are often associated with the uptake and transport of metal into the plant system. Broadly, aquaporin (AQP) family members are responsible for Al transport in plants [68].

Plants are capable of accumulating Al in above-ground tissues. Chenery, [69,70] classified plants as Al accumulators or non-accumulators after an investigation of 1000 plant species, plants that accumulate Al greater than 1000 mg Al·kg^−1^ in their leaves or roots were marked as Al accumulators and those with less than 1000 mg Al kg^−1^ were Al non-accumulators. Moreover, the author found that high Al accumulators were mostly woody plant species, wherein cereals were marked as lesser Al accumulators. For example, *Oriza sativa*, *Glycine max*, and *Zia mays* were found to accumulate less than 500 mg Al·kg^−1^ in the leaves. Rice accumulates less than 200 mg Al·kg^−1^ in shoots and is reported as a potential Al excluder [71]. In contrast, *Camellia sinensis* was recognized as the highest accumulator species that accumulated 13,500 mg Al kg^−1^ in the leaves. This result suggests that woody plants that normally thrive in soil with high concentrations of free Al have evolved internal mechanisms to cope with Al toxicity.

Al toxicity has dramatic impacts on plant growth and development that lead to significant yield reductions [9]. One of the clearest signs of Al toxicity is the inhibition of root growth in plants. Direct impacts of Al toxicity can be estimated by the evaluation of plant parameters based short and long term responses. Based on the result of several investigations, a variety of physiological, molecular, and economical occurrences resulting from Al toxicity have been detected in plants (Table 2). Moreover, it has been shown that the toxic Al tends to fix phosphorus (P) in a less available form which leads to a severe limitation of P availability for plant growth [13].Toxic Al leads to the production of ROS in the root apex, decreases root respiration, reduces polysaccharide deposition in the cell wall, reduces DNA replication by enhancing the rigidity of the double helix, induces programmed cell death, and results in a decline in photosynthetic efficiency [7,25]. These impacts of Al toxicity in plants can be induced within a few minutes to hours [14]. Rengel, [65] demonstrated that the first 15 min is the shortest critical time for the detection of measurable symptoms of Al-toxicity in intact root cells. The first symptoms of Al toxicity resulting in root growth inhibition were detected in wheat after 1 h of Al exposure [72]. Additionally, Al toxicity-induced disturbance of Ca^2+^ ion passing across the plasma membrane has been recognized as one of the earliest responses in wheat root apical cells.

The responses of plants to Al toxicity can vary across the species, variation can also be found among the different genotypes of the same species. Cereals along with other cultivated crop plants which diversely responded under Al stress can be classified as sensitive or moderate sensitive to Al toxicity. According to several investigations, the responses of various plants to Al exposure have been presented in Table 3. Among cereals, *Hordeum vulgare* and *Triticum durum* are considered the most sensitive crops to Al toxicity while *Oryza sativa* is reported as a potential Al excluder that significantly prevents Al toxicity compared to other cereals [71]. Notably, *Fagopyrum esculentum* and *Camellia sinensis* were able to store Al in above-ground tissue without symptoms of Al toxicity [9].

Al tolerance has been reported in a number of plants species enabling them to grow on acid soil. These tolerance mechanisms are mainly involved with the chelation strategy in roots by means of organic acids, such as citrate, malate, etc. Among the members, *ALMT1* located in root plasma membrane, was associated with Al tolerance through the exudation of malate into the rhizosphere in few plant species [73]. In addition, *NIP1;2* assists the Al-malate in *Arabidopsis* [68]. Yokosho et al. [74] demonstrated that *FRDL4* gene responsible for citrate efflux, was involved in citrate secretion from rice roots. Further, Al toxicity was alleviated in transgenic *Arabidopsis* roots while *Brassica oleracea* MATE (*BoMATE*) gene expression was more abundant in roots compared with wild-type plants. This gene was related to the of citrate exudation that confers Al tolerance in *Arabidopsis* [75]. 

Several reports have provided indications regarding the level of Al sensitivity in plants. It has been found that the inhibition of root growth occurs within in a few minutes to hours with a low concentration (µM) of toxic Al^3+^ [14]. Though in some cases low doses of Al have been reported to stimulate root and shoot growth of plants [3]. This might be due to Al enhancing capability of meristematic regions. Concentration-dependent Al toxicity and its effects have been widely manifested in various plants. For example, Al toxicity-induced rhizodermal cracks were observed in ahipa roots following exposure of roots to Al (11 µM) stress. In cowpea (*Vigna unguiculata*) the sensitivity threshold was observed at 0.1 µM Al wherein complete growth inhibition occurred at 40 µM Al [93]. Several sensitive and tolerant cereals and legumes with their responses to Al toxicity have been explored whereas the rice was marked as tolerant compared to other cereals [25]. Recently, nodulated legumes including common bean (*Phaseolus vulgaris*), soybean (*Glycine max*), and pea (*Pisum sativum*) has been reported to be sensitive to Al toxicity wherein their sensitivity doses were greater than 25 µM, 4.7 µM, and 50 µM, respectively, though soybean growth was inhibited at 10 µM Al [60].

## 4. Factors Affecting Aluminum Toxicity and Nutrient Imbalance

**Figure 1:**
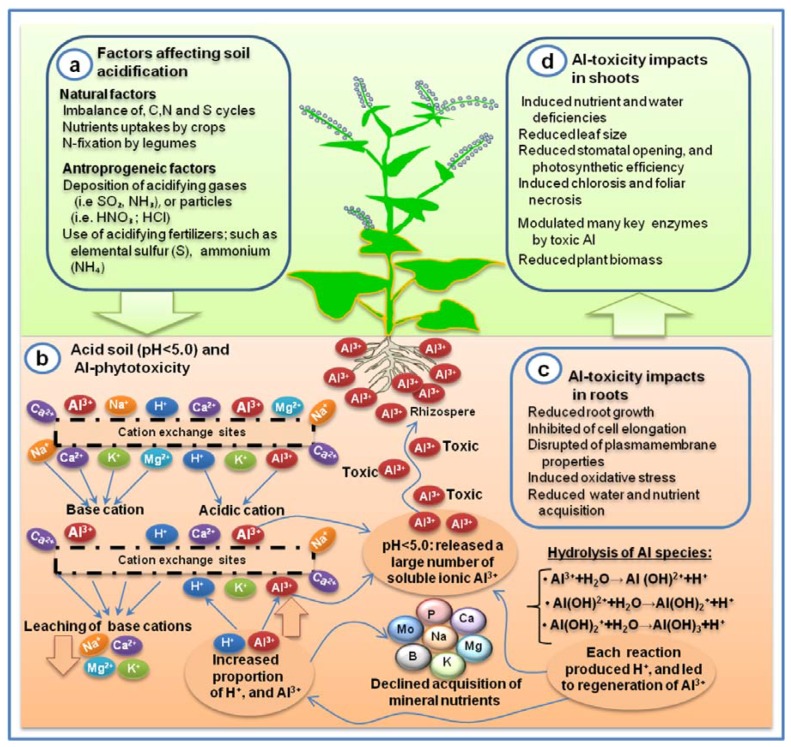
General overview of the factors affecting soil acidification, subsequent Al toxicity, and impacts in plants. (**a**) Natural and anthropogenic sources for occurring soil acidification. (**b**) Impact of cation saturation ratio in soil that led to increased exchangeable Al^3+^, and reduce acquisition of mineral nutrients. (**c**) Consequence of Al toxicity in roots, and thereby (**d**) impact of Al toxicity in shoots.

Soil acidification is an important factor that influences Al toxicity on agricultural land. The acidification of soil is caused by a number of natural and/or anthropogenic processes (Figure 1). Deposition of atmospheric gases or particles such as SO_2_, NH_3_, HNO_3_, and HCl; and application of acidifying fertilizer including elemental sulfur (S) or ammonium (NH_4_) salt accelerated the soil acidification process that led to increased soluble Al^3+^ concentrations in the soil solution [5]. Moreover, the imbalance of N, S, and C cycles, uptake of N by legumes, and intensified leaching of base cations (BC) were responsible for increasing H^+^ ions and decreasing soil pH level [94]. Over 100 years ago, it was noted for the first time that the concentration of soluble Al^3+^ increased in soils [95], this Al^3+^ was able to create phytotoxicity in the rhizosphere when the pH was below 5, and the most prominent sign of Al toxicity was considered the inhibition of root growth [62].

Acid rain/deposition has dramatic impacts on leachability of essential nutrient cations, mobility of toxic element (Al^3+^), and acidity development in soil [96]. Basic and acidic cations are available at soil exchange sites or in the soil solution. Cation exchange sites that hold cations in the soil are negatively charged. Soil is buffered during acidification by a series of chemical processes resulting in the replacement of exchangeable base cations (Ca^2+^, Mg^2+^, K^+^, and Na^+^) by H^+^ and Al^3+^ at the cation exchange sites [5]. Concurrently, the proportion of acidic cations such as H^+^ and Al^3+^ in the soil solution increases. Often, acid rain stimulates the leaching of base cations such as Ca^2+^, Mg^2+^, K^+^, and Na^+^ from soil. As a consequence, the essential nutrient cations such as Ca^2+^, Mg^2+^, and K^+^ are leached resulting in the depletion of base cations (BC) from the soil. The significant losses of these nutrient cations from soil solution or soil exchange sites result in nutrient imbalance in the soil. However, acid soils contain high amounts of Al^3+^ and a low amount of BC that are linked to deficiencies of important plant nutrients. Soil toxicity is known to be induced by the excess cations such as Mn^2+,^ Fe^3+^, H^+^ and Al^3+^ [3]. Among these, Al^3+^ is the most critical cation that leads to rhizotoxicity and severely impairs plant growth in acid soil. A ratio of BC/Al^3+^ less than 1 in soil solution was considered as a potential index for the adverse effect of soluble Al^3+^ and nutrient imbalance for plant growth [97]. Hence, the availability of BC and toxic Al^3+^ in acid soil depends on acid rain pH, soil properties, cation exchange capacity (CEC), soil texture and initial base content in soil [98]. 

Soil pH is often considered a master variable as it controls solubility, bioavailability, mobility, ionic speciation, and ultimately toxicity of any metal in the soil [99]. Ion availability in soil solution is influenced by low-pH. For example, Mn oxide solubilizes at soil pH below 5.5, and releases Mn^2+^ ions; at pH 4.3 a large amount of soluble Al^3+^ is released; at pH < 3.8 Fe becomes the most exchangeable ion in the soil solution [13,62]. The pH-dependent metal toxicity is quite complex; acid deposition to soil promotes soil acidity wherein more soluble ions are released into the soil solution. Consequently, potential phytotoxicity of metal ions was found to be enhanced due to their increased availability and concentration in soil solution [99]. The Al^3+^ and Al(OH)_4_^−^ are often described as the major rhizotoxic Al species at low and high-Ph levels, respectively. At low pH (about 4.3) soluble ionic aluminum (Al^3+^) is the most dominant form that is toxic for plant growth [3]. Surprisingly, it appears that toxic Al species not only inhibit root grow at pH 4.3 but also at pH 8.0, though the concentration of Al species at pH 8.0 was lower compared to at pH 4.3 [100]. Conversely, Al acquisition of root apices was higher at pH 8.0 compared to at pH 4.3. Calose (1,3-glucan) formation is often considered as the most perceptible indicator for Al toxicity that induced at pH 8.0, whereas the mobilization of callose was highly induced at pH 4.3 in the cortical region [100,101]. In addition, several nutrients such as P, K, Ca, Mg, Mo, and B contents were altered at low pH with Al toxicity [6,42]. So, the important note is that plants adapting to grow on low pH acid soils are threatened by the combination of Al toxicity and nutrient imbalance and/or deficiency.

The solubility of Al is an important factor that influences Al-availability, mobility, and toxicity in the environment [102]. Al was reported to highly soluble at more acidic (pH < 6.0) and at more alkaline (pH > 8.0) conditions but relatively insoluble at pH 6.0–8.0 [gibbsite; Al(OH)_3_] [103]. This insoluble form of Al is considered less toxic compared to soluble Al^3+^ [104]. Hence, Al species existing in clay fractions are mainly in a less toxic form such as alumino-silicate or aluminum oxide. Soil acidification promotes the process of its solubilization and mobilization which lead to potential phytotoxicity [105]. In acidic soil, when the pH drops (about 4.3) a large number of soluble Al ions (mostly Al^3+^) are released, this toxic Al^3+^ rapidly inhibits root elongation [62,100]. At neutral pH (7.0), Al hydroxide species (e.g., AlOH_3_) are relatively insoluble but at pH above 7.5, Al species are formed as Al(OH)^4−^ and solubilized again [61]. However, a relationship has been observed between pH and Al solubility, wherein the Al solubility increases when pH is below 4.5 in acid soil [3]. In addition, Al toxicity is also known to be stimulated by the pH-dependent hydrolysis intensity of several Al species in soil solution [6]. Hydrolysis of ions occurred when the charge/radius (z/r) ratio is large enough to break down the bond (H–O), and releases H^+^ into the soil solution. pH-dependent hydrolysis of mononuclear Al species are presented by simple equations (Figure 1), wherein each chemical reaction indicated the production of H^+^ that leads to the generation of more soluble Al^3+^ Hence, the hydrolysis of these Al species occurs at low pH (<5.0).

Toxic-Al interferes in the acquisition, accumulation, localization, and utilization of most of the mineral elements. For instance, the uptake of mineral nutrients such as Ca^2+^ (69%), Mg^2+^, K^+^ (13%), and NH_4_^+^ (40%) were inhibited by Al toxicity, and Al was known to be enhanced influx of the specific anions such as HN_3_^−^ (44%), and PO_4_^3−^ [42]. Al toxicity-induced nutritional imbalances have been widely manifested in several plant species. For instance, a distinct Al accumulating pattern with nutritional imbalance (e.g., Ca, Mg, P, and K) has been detected in eleven pteridophytes families [106]. Al toxicity generally inhibits to uptake up macro-and micro elements plants, whereas tolerant cultivars found to be exhibited several macro elements including Ca and Mg [12]. In wheat, both sensitive and tolerant genotypes manifested a marked reduction of K and Mg content whereas the concentration of Ca, Al and Si increased in roots [78]. Hence, the sensitive genotypes exhibited higher Al accumulation and nutritional imbalance in both roots and shoots than the tolerant one. 

Al-toxicity reduced K, Mg, Ca, and P accumulation in two contrasting rice cultivars, whereas the utilization of P, Ca and Mg increased more in tolerant cultivar than a sensitive plant [107]. Simon et al. [108] reported that Al exposure was involved in the reduction of Ca, K, Mg, Mn, Fe and Zn contents in tomato. Zobel et al. [109] observed that the changes in fine root diameter with changes in nutrient such as N, P, and Al in plants. Additionally, the *specific absorption rate of B* (SAR_B_) was significantly influenced by Al-toxicity. Poschenrieder et al. [110] detected a correlation between the Al-induced reduction of B absorption and the root growth inhibition in maize. From the above-mentioned literature it appears that Al toxicity induces the imbalance of nutrient uptake and acquisition in plants

## 5. Role of Mineral Nutrition for Mitigating Aluminum Toxicity in Plants

A large number of strategies have been explored to mitigate the Al toxicity in plants to acid soils. Most of the strategies can be divided into two classes: inorganic amendments such as exogenous application of mineral elements including Ca, Mg, P, S, B, and Si and ground oxide/hydroxide [111,112,113,114,115,116,117] (Table 4 and Table 5) and organic amendments such as organo-mineral fertilizers, plant growth promoting bacteria (PGPB), green waste compost, plant-derived biochars, and their combination with other minerals [118,119] which played a pivotal role in Al toxicity alleviation in plants to acid soils. Conversely, we did not observe considerable existing literatures concerning the role of K, Mn, Zn, and Mo in Al toxicity alleviation in plants though these are essential minerals. The following sections clarify the role of major nutrients based amendments to mitigate Al toxicity in plants to acid soils.

### 5.1. Calcium (Ca)

Ca alleviates Al-induced rhizotoxicity in different crops growing in acid soils [111]. Ca was reported to alleviate Al toxicity through several mechanisms: (i) displacement of Al from the cell membrane surface (CMS) by an electrostatic effect; (ii) restoration of Ca^2+^ on the CMS; and (iii) ionic interaction between Ca^2+^ and Al^3+^ that occurred at cell surface and at Donnan free space (DFS) of root cells [120,121]. Kinraide, [121] reported that 1 mmol·L^−1^ concentration of Ca is capable to inhibit the effect of 1 µmol·L^−1^ Al. Ca nutrient was known to be applied as different practical alternatives such as liming oxide (CaO), hydroxide [Ca(OH)_2_], calcites (CaCO_3_), dolomites [CaMg(CO_3_)_2_], and gypsum (CaSO_4_) including its bio-products. Application of these Ca-amendments greatly influences the effectiveness of Ca for mitigating Al toxicity. Moreover, the application of Cd before Al stress is more effective to mitigate Al-induced damage than that of Cd supplementation during Al treatment [122]. In the following sections, we discuss the beneficial effects of several Ca-amendments such as lime (e.g., calcite, dolomite), phosphogypsusm (PG), and gypsum (G) with various application doses on different crop plants to acid soils.

#### 5.1.1. Liming

Liming is an important and well-known approach for correcting acid soil, along with restoring Ca availability, and alleviating Al toxicity in plants [5,136]. Application of Ca or Ca-Mg containing minerals [calcites, CaCO_3_; dolomites, CaMg(CO_3_)_2_] to acid soil increases the pH level and reduces toxic Al concentration [137]. Consequently, enhances the cation exchange capacity (CEC), and P availability by inactivation Al and Fe of soils. Several factors such as neutralizing value (NV) or purity of lime, particle fitness (PF), and lime distribution (LD) indicate the effectiveness of lime [136]. NV of agricultural lime materials is determined by a percentage (%) relative to the neutralizing value of standard CaCO_3_, and is known as *calcium carbonate equivalent* (CCE). It has been marked that calcites and dolomites consist of 70–100% and 70–109% CCE, respectively [136]. PF is important for lime effectiveness because large particles (>1.7 mm) can remain unreacted in the soil for many years. Moreover, limes are known to be extended about 3 mm after placing, and takes more time to soluble, and subsequent neutralization of soil acidity. Therefore, it should be distributed by the following incorporation at least two months before planting. 

#### 5.1.2. Phosphogypsum (PG)

PG is primarily calcium sulfate hydrate formed as a by-product from the industry of phosphate fertilizer [138]. It is mainly composed of Ca^2+^, SO_4_^2−^ and a small amount of phosphorus (P), silicon (Si), and fluoride (F) [139]. PG was known to be involved in Al toxicity alleviation by complexing with F at low pH. Several reports have provided indications regarding the use of PG as an alternative to Ca and SO_4_^2−^, applied for balancing acidity and alleviating Al toxicity in acid soils [140,141] (Table 4). Mays and Mortvedt, [142] suggested a dose range from 500–1000 kg·ha^−1^ for a single PG application. Conversely, a combined PG with CaCO_3_ amendment, and/or gypsum (2500 kg·ha^−1^) treatment was found to reduce Al^3+^ concentration at a depth of up to 5 cm in soil, also increased root density and decrease Al^3+^ concentration in apple trees [125].

#### 5.1.3. Gypsum (G)

G is known as a soft sulfate mineral composed of a calcium sulfate dihydrate (CaSO_4_·2H_2_O), widely used as an amendment in soils under acidic conditions. The solubility of G (2.5 g·L^−1^) was reported approximately 5-fold higher than calcite lime (0.5 g·L^−1^) [143]. G is an important source of Ca and S which improves mineral (N, P, K, Mn, and Zn) profiles in plants [144]. Several calcareous amendments would offer a suitable option for decreasing Al toxicity and soil acidity [145] (Table 4). To reduce Al toxicity the role of SO_4_ is important in acid sub-soils, wherein Al complexes organic ligands (OL). Complexing OL after G application has been detected in Malaysian acid soils resulting in increased AlSO_4_^+^ and decreased Al activity that led to enhanced corn yield [146]. Interestingly, the toxic effects of Al species [Al^3+^, Al(OH)^2+^, and Al(OH)_2_^+^] have been alleviated by G whereas soybean roots were elongated at 500 mM G application [111]. 

G not only alleviates Al toxicity but also provides nutritional benefits in plants grown in acid soils. For instance, N, P, K, S, Mn and Ca levels increased in blueberry leaves after a combined G (4.0 t·ha^−1^) and NPK fertilizer (0.3 t·ha^−1^) application to acid soil, and enhanced stem elongation, bud survival, and blossom quantity [147]. Korcak [148] found that soil pH and Ca levels were increased after G (0.60 t·ha^−1^) application for five years, whereas Ca levels were inconsistent in leaves and fruits. Recently, Tirado-Corbalá et al. [131] declared that G is able to provide Ca and S benefits to nutrient-limiting soils that led to enhance soil fertility and alfalfa growth. Moreover, the authors undertook a long term (12 years) study on alfalfa with G (22.2 t·ha^−1^) application (Table 4).

### 5.2. Magnesium (Mg)

Mg is an essential nutrient that plays the vital role in phloem loading of sucrose, also alleviates soil-borne Al toxicity [149]. Unfortunately, Al toxicity induces Mg deficiency that leads partitioning of dry matter and carbohydrates between roots and shoots [150]. Consequently, observed that plant growth and yield were severely impaired in Mg-deficient plants. Therefore, exogenous application of Mg would be a suitable approach to alleviate Al toxicity in plants. Exogenous application of Mg found to be alleviated Al toxicity through (i) increasing ionic strength of the solution [120]; (ii) reducing Al saturation at the space outside of plasma membrane (PM), declining Al activity at PM of root cells [151]. Bose et al. [152] also elucidated that several mechanisms relevant to Mg mediated alleviation of Al toxicity in plants. For example, intercellular Mg-dependent regulation of organic acid anions (OAs) exudation, stimulation of *H^+^-ATPase* activity, modulation of free cytosolic Ca^2+^ spikes, and regulation of ROS homeostasis under Al toxicity in plants.

Exudation of OAs one of the best strategies for phytotoxic Al exclusion in several plant species to acid soils [13]. Exogenous application of Mg (50 µM) to medium ameliorated Al toxicity by enhancing citrate exudation in soybean [113]. Interestingly, Mg pre-treated soybean seedlings led to induce citrate secretion within 1 h following Al exposure. *H^+^-ATPase* is a key protein known to be involved in nutrient acquisition [153], stomatal opening [154], auxin transport and cell elongation [155]. It has been reported that *H^+^-ATPase* activity inhibited by Al toxicity in *Vigna umbellata* roots, surprisingly *H^+^-ATPase* activity was marked to be rebooted and induced citrate exudation while added 10 mM Mg to the same nutrient solution [156]. Similarly, exogenous application of 20 mM Mg treatment following Al exposure stimulated *H^+^-ATPase* activity, along with enhanced citrate exudation that alleviated Al toxicity in *Vicia faba* [157]. In a plant cell, free cytosolic Ca^2+^ found to increase during Al stress, and free cytosolic Ca^2+^ activity usually maintained 100–200 nM range, over this dose might be induced cytotoxicity in the cell, also observed free cytosolic Ca^2+^ level significantly increased in yeast cells due to exclusion of Mg nutrition [158]. Additionally, it has been reported that elevated Mg modulates intracellular Ca^2+^ permeable channels, and along with lead to release free Ca^2+^ from the internal organelles [159]. Therefore, it is hypothesized that the Al exposure in plants enhanced Mg content that leads to prevent Ca^2+^ cytotoxicity.

Several studies have provided indications regarding the Mg nutrition not only alleviates Al toxicity but also enhances root growth in diverse plant species. Root growth was found to be enhanced by Mg nutrition (10–200 µM treatment) in several legumes such as soybean (*Glycine max*) [113], rice bean (*Vigna umbellata*) [156] and broad bean (*Vicia faba*) [157]. Moreover, Mg transporter genes were associated to ameliorate Al toxicity in plants. Overexpression of an Mg transporter gene (*AtMGT1*) alleviated Al toxicity by enhancing Mg acquisition in *N. benthamiana* [160]. Al treatment (50 µM AlCl_3_, at pH 4.2) in nutrient solution showed an increased Mg uptake and free Mg concentration in the cytoplasm of Al-tolerant *Arabidopsis* (*Col-0* and *alr104*) than sensitive one (*als-5* and *als3*) [152]. Such elevation of cytosolic Mg nutrition might be a key indication of a tolerant plant to alleviate Al toxicity. 

### 5.3. Phosphorus (P)

P is an essential macro-mineral that plays an important role in plant growth and development under normal and/or stress conditions [161,162]. In acidic soils, inorganic phosphate (Pi) is fixed by Al/Fe, it becomes critical when the pH drops; the result is a severe limitation of Pi in acid soils [13]. Consequently, plants surviving in acid soils have to face both Al toxicity and P deficiency. Exogenous application of P alleviates Al toxicity in a number of plants such as sorghum [163], buckwheat [164], and wheat [165] on acid soils. Tan and Keltjens, [163] found that plant biomass production was not influenced by a low concentration of Al (0.4 mg·L^−1^), but plant growth and dry matter yield (DMY) were severely inhibited at a high Al concentration (1.6 mg·L^−1^) in sorghum. In this regard, the addition of P alleviated Al toxicity by increasing root respiration and nutrient uptake that led to enhanced DMY. Iqbal [165] observed that P content in wheat seedlings was largely reduced by Al stress (150 mg AlCl_3_ kg^−1^ soil), conversely pH level was found to be balanced and increased P level after addition of exogenous P (160 mg P kg^−1^ soil) to soil. Chen et al. [166] provided a threshold of P alleviating Al toxicity based on tested plants, and mentioned if the value of P/Al molar ratio exceeds 5 in the root cells, that plant can alleviate Al toxicity. Recently, P application (8 g·plant^−1^) in nursery conditions was reported to enhance plant growth, height, root collar diameter, and chlorophyll content in maple tree [161]. 

Several effective strategies have been explored for plant production under Al toxicity and P deficiency in acidic soils. Ch’ng et al. [167] suggested to increase P availability by adding organic amendments (e.g., biochar, compost etc.) as he observed the total P and Pi were increased by organic amendments that effectively fixed toxic Al and/or Fe in acid soil. In addition, several molecular breeding related strategies have been explored in plant response to Al toxicity and P deficiency. Sasaki et al. (2004) cloned malate transporter *TaALMT1* gene from wheat and transferred them into Al-sensitive barley that obtained the trait of Al-resistance. Wang et al. [168] suggested a ‘*root breeding*’ approach to developing P-efficient plants based on those that have the ability to utilize native and exogenous P from acidic soils. Recently, Chen and Liao [13] highlighted the opportunity for organic acid synthesis in crop production for sustainable agriculture though genetic manipulation as the application of P-fertilizers are costly and can be seen to be environmentally risky. 

### 5.4. Sulfur (S)

Until the 1970’s, S was mainly regarded as a neglected element in soil science, though it has a pivotal role in the production of protein, vitamins, chlorophyll, and glucoside oil in plants [169]. Recently, S has received more attention due to its capacity to modify metal toxicity as well as having a vital role in plant growth and development [116,170,171]. Several studies have provided evidence that S-containing components alleviate Al toxicity in wheat [172], barley [173], oilseed rape [174] and citrus trees [116]. In the above studies S exerts protective functions against Al toxicity through: (i) increasing antioxidant activity, and decreasing ROS and lipid peroxidation levels; (ii) decreasing uptake of Al in roots and shoots; (iii) increasing uptake of several nutrients viz phosphorus (P), magnesium (Mg), and calcium (Ca); and (iv) enhancing Al-induced secretion of organic acid anions (OAs) from plant roots. The toxicity induced by several heavy metals was alleviated in plants by exogenous S addition, though the efficiency of alleviation mostly depends on the S-application strategies, doses, and sources. Generally, high doses of S are recommended to abate arsenic (As) uptake in plants. For example, S treatment (120 mg S·kg^−1^ soil; Na_2_S_2_O_3_·5H_2_O) was applied to As (20 mg As·kg^−1^ soil; Na_2_HAsO_4_·12H_2_O) contaminated soil, and it was found that As concentrations reduced in rice grains by 44% compared to grains from the treatment without S application [175]. Recently, S addition (0.5 mM S; MgSO_4_ and/or 0.5 mM Na_2_SO_4_) was shown to be alleviated Al toxicity by increasing minerals (P, Mg and Ca) and relative water contents; decreasing Al and H_2_O_2_ contents, and involving S-metabolism and antioxidant enzymes in citrus [116]. Additionally, several studies have reported that S increased mineral components that supported to alleviate several metal toxicities, along with Al toxicity in plants [116,170], and better increased NO_3_^−^ and NH_4_^+^ levels in the soil compared to an NPK fertilizer treatment [176].

### 5.5. Boron (B)

B is an essential micronutrient which was reported to decrease the accumulation of toxic Al in several plants [114]. Hossain et al. [177] conducted a nutrient culture to assess the impact of B application (200 µM) on Al toxicity (50 µM Al; pH 4.5), B deficiency, and growth of 15 wheat cultivars. Subsequently, they found that malate exudation level increased in the roots of all cultivars under Al toxicity (100 µM Al; pH 4.5). Among the 15 cultivars, the most tolerant cultivar exuded approximately 6-fold higher malate content than a sensitive one. Additionally, vigorous seedling growth was observed at 40 µM B application, and 200 µM B alleviated Al toxicity in a sensitive wheat cultivar. Recently, Riaz et al. [178] found that B treatment (25 µM B as H_3_BO_3_) alleviated Al toxicity by: (i) improving the activities of antioxidant enzymes such as peroxidase, catalase, and ascorbate peroxidase, and; (ii) reducing mobilization of toxic Al in roots and shoots of rapeseed. Gupta et al. [179] demonstrated that B concentrations (20–100 µg B·g^−1^ DW, DW = dry weight; 20–100 µg boron present in per gram dry weighted sample. Either root or shoot sample.) were adequate for growth, but an excess level of B (200 µg B·g^−1^ DW) was associated to induce toxicity in plant cells.

### 5.6. Silicon (Si)

Si is the second most abundant metal in the earth’s crust [180]. In soil medium, plants take up and accumulate Si ranging from (1–100 g Si·kg^−1^ DW) through different modes, uptake, and transport processes [181]. Si is taken up in plants as orthosilicic acid (H_4_SiO_4_), and rice is considered as typical Si accumulator (100 g Si·kg^−1^ DW) [182]. Si is still to be considered as an essential element, although the beneficial impact of Si concerning multiple stress tolerances in plants has been wildly recognized [183]. Several studies have demonstrated the effectiveness of Si application towards decreasing Al uptake by plants grown hydroponically or in pot experiments [135,184]. Singh et al. [135] demonstrated that exogenous Si addition alleviated Al toxicity by reducing Al accumulation, and preventing the Mg and Zn deficiency in rice during Al stress. Interestingly, Si showed efficiency by forming Al-Si complexes in sorghum mucigel and outer cellular tissues. Consequently, this Al-Si complex inhibited the binding of toxic Al to the cell wall [185]. Recently, a novel approach has been applied to alleviate Al phytotoxicity in wheat through silicon (Si)-rich biochar amendment in soil [184]. In the same study, the authors suggested this cost-effective approach as it ameliorated the acidic soil, subsequently alleviated phytotoxicity by a mechanism wherein Al involves the chelation of metal with Si-biochar (Al-Si). Additionally, Si-particle reduced the amount of soil exchangeable Al and prevented the migration of toxic Al in different plant tissue. 

### 5.7. Miscellaneous 

Several low-cost effective and available ameliorants such as alkaline slag (AS) coal fly ash (CFA), and red mud (RM), are known to be involved in correcting soil acidity, and subsequent alleviation of Al toxicity under tea plantation [186]. In addition, it was found to be increased soil pH and cation exchange capacity (CEC%), and decreased exchangeable Al by RM, AS, and CFA. A mechanism by which these amendments were involved in “*formation and retention of hydroxyl-Al-polymers*” that alleviated Al phytotoxicity. The important note is that constitutes of these byproducts used in agriculture with limited environmental hazard. These industrial byproducts provided a good source of base cations (Ca^2+^, Mg^2+^, K^+^, and Na^+^) and anions (P and S). Therefore, use of these industrial byproducts would be effective as an alternative source for balancing soil acidity, and alleviating Al phytotoxicity instead of the traditional use of lime and gypsum.

Hormones, organic acids, and polyamines participated in favor of alleviation of Al toxicity in different plants [3,13,187]. The foliar application of *indole acetic acid* (*IAA*; 25 µM) reduced the accumulation of Al in the root apex in wheat [188], and *IAA* (6 mg·L^−1^) alleviated Al-induced damage of cell structure in alfalfa [189]. *IAA* stimulated the plasma membrane *H^+^-ATPase* activity and reduced Al contents in root tip, cell wall, and pectin fractions. This process participated in Al-toxicity alleviation by decreasing toxic-Al binding ability in pectin through enhancing H^+^ secretion in rhizosphere. Polyamines (*PAs*), and salicylic acids (*SAs*) are involved in organic acids (*OAs*) secretion, osmolyte biosynthesis, and activation of the antioxidant system in plants [3,190,191]. Putrescine (*Put*) is an important signaling molecule; Al toxicity-induced root inhibition was alleviated by *Put* through reducing ethylene synthesis [192]. It has been observed that Al-exclusion and internal Al toxicity alleviation mechanisms rely on Al-induced secretion of organic acids anions and phenolic compounds in root apex [193]. SA alleviated Al toxicity by positive modulation of the citrate efflux in *Cassia tora* roots [194]. In addition, Al toxicity was alleviated in tomato [195], and soybean [191] by SA (<20 µM) through activation of the antioxidant system. 

Biofertilizers (e.g., micorrhizal fungi (MF), plant growth promoting bacteria) and biochars (e.g., agricultural wastes, organic wastes) application provide several advantages to overcome Al^3+^ toxicity in plants to acidic soils. In acidic soil (pH 3.5) with Al stress (1.0 mM; AlCl_3_·6H_2_O), following the inoculation of arbuscular mycorrhizal species: *Rhizophagus irregularis* (formerly *Glomus intraradices*) and *Funneliformis mosseae* (formerly *Glomus mosseae*) supported to enhance plant yield, total biomass, and better nutritional (N, P, K, Ca, Mg, Fe, Zn and B) status in *Cucurbita pepo* compared to non-inoculated plants [196]. In addition, application of arbuscular mycorrhizal inoculum *Glomus intraradices* alleviated Al toxicity by decreasing toxic Al accumulation, and enhancing Ca, Mg and P accumulations in banana roots [197]. 

Plant growth promoting bacteria (PGPB) played an important role against Al^3+^ toxicity and soil acidity. In field condition, 4 t·ha^−1^ biofertilizer (a consortium of PGPB: *Bacillus* sp., *Stenotrophomonas maltophila*, *Burkholderia thailandensis*, and *Burkholderia seminalis*; 5 × 10^9^ CFU·g^−1^ bacterial cells) combined with ground magnesium limestone (GML) application ameliorated Al^3+^ toxicity in rice to acid sulfate soil [119]. A mechanism was involved wherein PGPB-mediated Al toxicity amelioration occurred by chelation of Al-organic acids (Al-OAs). Concurrently, PGPB-led to the production of phytohormones which were associated with enhancing better growth and yield in rice [118,119]. Recently, the promising role of rice straw derived Si-rich biochars has been explored toward the alleviation of Al-toxicity, and soil acidity [184]. Moreover, an alleviation mechanism of Al phytotoxicity was involved in wheat wherein biological sourced-Si coordinated simultaneously with Al to form Al-Si complex for toxicity alleviation, on other hand Si-particles prevented the migration of toxic Al through root tips cell. 

## 6. Conclusions and Prospects

This extensive review clarified that Al toxicity can be effectively mitigated in plants by the application of nutrient elements in optimum quantities. Exogenous phosphorus (P) application is more beneficial during plants were found to be affected rigorously by soil acidification, Al toxicity and P deficiency. P was able to repair P-deficiency in acid soil, increased root respiration, plant growth, chlorophyll content, and dry matter yield. Ca amendment (liming) is more effective for correcting soil acidity and alleviating Al toxicity, but it takes more time to solubilize, and neutralize soil acidity. Moreover, continuous input of P-fertilizer, and lime in soil is expensive and environmentally risky. Gypsum (G) is attributed with better solubility (approximately 5-fold higher than lime), supplied dual benefits simultaneously as an alleviator of Al toxicity, and inducer of nutrients content. Magnesium (Mg) is effective for decreasing Al-activity at plasma membrane of root apex, and able to prevent Al toxicity-induced Ca cytotoxicity. Sulfur (S) can be used as several metals (Al, As) toxicity alleviator. The beneficial impact of exogenous Si-application has been widely recognized against Al toxicity in plants though it is still to be reconsidered as an essential element. Despite these above-mentioned facts, less attention has been given to clarify the potential roles of exogenous application of K, Mg, Zn, and Mo toward Al toxicity alleviation in plants to acidic soils. 

Some other promising approaches have been applied considering miscellaneous mineral elements to ameliorate Al toxicity in plants to acid soils. For example, exogenous application of several low cost effective industrial byproducts (alkaline slag, coal fly ash, and red mud), hormones (auxin; *IAA*), organic acids (OAs; *citrate*, *oxalate*, and *malate*), polyamines (*putrescine*), biofertilizers (e.g., micorrhizal fungi, growth promoting bacteria), biochars (e.g., agricultural wastes, organic wastes) mitigated Al toxicity in plants to acidic soils. However, this review presents existing knowledge concerning the application of mineral nutrition on Al toxicity mitigation in plants that could be a potential approach for further crop plants improvement under Al toxicity and soil acidity worldwide.

## Figures and Tables

**Table 1:** Concentration of Al (g·kg^−1^) in soil in different countries.

Country	Area/Region/Location	Nature/Type of Soil	Soil Depth (cm)	Range of pH	No. of Samples	Range of Al Concentration (g/kg)	Mean	References
Bangladesh	Dinajpur, Rangpur, Bangladesh	Paddy soil; sandy loam, non-calcareous, acidic-alluvial	0–15, 15–30	4.8–5.4	04	0.024–0.059	0.043	[43]
	Hill of Chittagong University, Bangladesh	Hill topsoil; surface, subsurface	0–12	4.4–5.5	45	0.036–0.058	0.048	[44]
Brazil	Cantareira State Park, Brazil	Forest back-ground	0–20, 20–40	3.3–5.7	11	17.1–71.8	41.1	[45]
China	Guizhou province, China	Yellow-brown	0–30	3.5–4.8	13	65.2–128.5	106.4	[46]
	Sichuan, Zhejiang, and Jiangsu, Southeast China	Original, bulk and rhizosphere soil	0–20	3.4–5.9	18	0.18–0.58	0.37	[47]
	Central and Southwest China	Yellow sandstone, red earth, and Pleistocene deposits	0–20	3.5–5.7	12	75.5–108.3	89.2	[48]
Canada	Northwestern Alberta, eastern and western Canada	Podzolic, luvisoli, gleysolic, subsoil	-- --	3.5–5.4	35	0.015–0.027	0.069	[49]
Czech Republic	Jizera Mountains area, Czech Republic	Horizon; Organic-fragmented organic-humified organic-mineral mineral	0–12.2	3.8–4.2	491	0.0057–0.017	0.0097	[50]
France	Vosges Mountains area, North Eastern France	Acid brown, podzolic	0–220	3.7–4.6	8	0.0003–0.047	0.0173	[51]
India	Bihar, India	Fine mixed, fine loamy, sandy mixed	0–150	4.4–7.0	13	0.0003–0.0051	0.0023	[52]
Ireland Republic	South-eastern region, Ireland	Grassland, tillage, forest, peat	0–10	5.3–5.9	295	0.0–89.1	45.6	[53]
Japan	Hokkaido, Tohoku, Kanto, Kinki, Hokuriku-Chubu, Chugoku, Shikoku, Kyushu, and Okinawa	Agricultural soil; paddy/upland field	0–15	-- --	180	33.0–117.0	79.0	[54]
Korea Republic	Osan, Korea	Sandy clay loam	-- --	4.0–7.1	-- --	0.384–1.825	0.836	[55]
	Seoul, Ulsan, Hongchon, Korea	Forest; urban and industrial areas	0–15	4.1–4.3	-- --	-- --	-- --	[56]
New Zealand	North Canterbury, Central Canterbury, Central Otago	Stony brown, brown, dense brown hill soil	0–15	4.9–6.7	14	0.0005–0.0174	0.0057	[57]
USA	Palouse Conservation Field Station (PCFS) located near Pullman, WA, USA	Agricultural soils; silt loam	0–30	4.7–6.3	80	0.034–0.055	0.0196	[58]
	Crawford County, OH, USA	Subsoil, acid soil	0–110	4.5–7.5	99	0–0.25	-- --	[59]

**Table 2:** Impacts of aluminum toxicity in plants.

Index of Toxicity	Sensitivity/Time of Al Exposure	Impacts of Al Toxicity	Conditions of Experiment	Aim of Assessment	References
Grain yield	Low/LD	Al-induced delay flowering; significantly reduced grain yield in sensitive cultivar	NS	FS	[76]
Biomass production	Moderate/LD	Reduced dry matter yield	NS	FS	[77]
Nutrient imbalance	Moderate/SD	Resulted imbalance of macronutrients including Mg, K and P	NS	TS	[78]
Survival	Low/LD	Resulted dry weight is the most sensitive tolerance index while survival is considered as the most cost-effective indicator of tolerance	SC	TS	[79]
Root growth	High/SD	Root growth was significantly inhibited by toxic Al ions in acid soil	SC	TS	[80]
Change of root system	High/LD	Indicated potential toxicity problem; root tips and lateral roots become more stubby, turned brown, and inhibited fine brunching	SC	TA	[81]
Rapture/cracks of root	High/SD	Induced rhizodermal cracks on roots after exposures to Al (11 µM)	NS	TA	[82]
Plasma membrane (PM)	High/SD	Al^3+^ ion attached to PM; cells become more leaky and rigid	NS	TS	[83]
Cell division and elongation	High/SD	Al resulted the disordered the arrangement of the cells, deformed cell shapes, altered cell structure, and the shorter of the meristematic zone of the root tips	NS	FS, AR	[23]
Interference with enzymes	Moderate/SD	Down regulated peroxidase and chitinase isoforms in root tips	NS	FS, TS	[84]
Organic acid exudation	Low/LD	Organic acids (oxalate, citrate) enhanced plant growth and adaptation following Al stress to acid soil	SC	FS, AR	[85]
Callose formation	High/SD	Resulted a link between callose formation and Al-induced inhibition of root growth	NS	TA, TS	[86]
Auxin transport	High/SD	Al toxicity inhibited (*IAA*) transportation from the shoot base to root tip; though exogenous application of *IAA* alleviated Al stress	NS	FS, AR	[23]
Al content in pectin	High/SD	Aggravated Al^3+^ toxicity due to accumulation of more Al in pectin	NS	TA	[87]

Plant-based indices for assessment of the impacts of Al-toxicity (LD, long-duration; SD, short-duration; FS, fundamental study; AR, applied research; TA, toxicity assessment; TS, tolerance screening; NS, nutrient solution; SC, soil condition).

**Table 3:** Sensitivity of plants to aluminum toxicity, modified from [3,25,60,82,88,89,90,91,92].

Sensitivity Threshold		
High Sensitive (Indicator Plants)	Moderate Sensitive	Low Sensitive (Resistant/Tolerant Plants)
*Hordeum vulgare* L. (barley)	*Rapanus sativus* L. (radish)	*Fagopyrum esculentum* Moench (buckwheat)
*Triticum aestivum* L. (wheat)	*Sorghum bicolour* L. Moench (sorghum)	*Camellia sinensis* L. (tea)
*Triticum durum* Desf. (durum wheat)		
*Lactuca sativa* L. (lettuce)	*Capitata* var. alba L. (cabbage)	*Oryza sativa* L. (rice)
*Beta vulragis* L. (beet)		
*Phleum pretense* L. (timothy-grass)	*Avena sativa* L. (oat)	*Zea mays* L. (maize)
*Glycine max* Merr. (soybean)		
*Pisum sativum* L. (pea)	*Medicago sativa* L. (alfalfa)	*Brassica rapa* L. (turnip)
*Phaseolus vulgaris* L. (common bean)		
*Pachyrhizus ahipa* Wedd. (Parodi)	*Secale cereale* L. (rye)	*Agrostis gigantean* Roth. (redtop)

**Table 4:** Calcareous amendments-induced alleviation of Al toxicity in plants to acid soils.

Amendments	Plant Species	Dose (t·ha^−1^) and Duration	Outcomes	References
Phosphogypsum (PG)	*Zea mays* L., *Triticum aestivum* L., *Glycine max* L.	12.0, (0–8.0 cm), 180 days	Increased Ca concentrations in all crops; enhanced root growth; provided nutrients to the soil and reduce Al^3+^ activity.	[123]
	*Medicago sativa* L. cv. Hunter River and *Glycine max* L. Merr. cv. Lee	2.0, (60–80 cm); 50 days	Ameliorated subsoil acidity; Increased Ca in soil solution; Reduced exchangeable Al, and increased in crop growth.	[124]
	*Malus domestica* Borkh. cv. Gala/MM 106	2.0, (40–60 cm), 90 days	Decreased exchangeable Al; increased Ca in topsoil as well as plant leaf; increase root density.	[125]
	*Trifolium* spp.	2.5, (0–25 cm), 1.5 years	Reduced toxic Al concentration in soil solution and exchange site in subterranean clover-based pasture.	[126]
	*Zea mays* L.	34.9% PG; (5 × 7 × 5 cm)	Reduced soil acidity; decreased Al activity of in the soil solution; increased root development.	[127]
Gypsum (G)	*Lolium perenne* L. cv. Nui	2.0, (0–20 cm)	Balanced nutrient elements; increased forage quality and yield; reduced exchangeable Al; increased soil pH.	[128]
	*Vaccinium corymbosum* L.	4.4, 60 days	Enhanced Ca and S contents; reduced Al concentration and lipid peroxidation in roots and leaves; Ameliorated Al toxicity in plant by gypsum.	[129]
	*Arachis hypogaea* L.	1.0, 60 days	Reduced Al phytotoxicity; increased nodules, pods, and yield of groundnut.	[130]
	*Medicago sativa* L.	22.2, (0–75 cm), 12 years	Provided Ca and S benefits as a source in nutrient-limiting soils; enhanced soil fertility and alfalfa growth.	[131]
	*Zea mays* L.	11.0, (40–60 cm), 1 year	Improved root density; Increased Ca, Mg, and SO_4_-S; declined the level of exchangeable Al.	[132]

**Table 5:** Mineral nutrition induced protection in plants under Al toxicity.

Plant Species	Dose and Duration of Al Exposure	Mineral Treatment	Plant Responses	References
*Glycine max* (L.) Merr.	2 µM Al, 24 h	500–2000 µM Ca, 3 days	Mitigated the inhibition of root growth and root hair formation during Al toxicity; enhanced protection against the deleterious effect of toxic Al in soybean.	[111]
*Triticum aestivum* L.	20 µM Al, 28 days	1600 µM Ca; 800 µM Mg, 7 days	Ca and Mg alleviated Al toxicity in wheat, respectively; decreased Al^3+^ activity at PM surface in root cell.	[112,133]
*Glycine max* (L.) Merr.	100 mM Al, 72 h	25 µM Mg, 3 days	Increased root growth; Alleviated Al rhizotoxicity during lateral root elongation.	[113]
*Eucalyptus grandis* × *E*. *urophylla* clones “G9”and “DH32-29”	5 mM Al, 20 weeks	200 µM P; 140 days	Al resistant G9 clone, enhanced more malate, oxalate, and citrate secretion in roots; P was involved in elemental Al fixation in roots, and restricted Al transport to the stems and leaves; Increased the activities of *PEPC*, *CS* enzymes.	[117]
*Citrus grandis*	1 mM Al, 16 days	0.5 mM S, 126 days	Reduced Al transport in roots, shoots and leaves; Decreased H_2_O_2_ production; Increased, P, Mg, Ca and RWC; Enhanced OAs in roots.	[116]
*Citrus grandis*	1.2 mM Al, 18 weeks	20 μM B, 126 days	Over 100 genes including *GSTZ1, TRX-M4, CLM 19*, *IAA-amino acid hydrolase ILR1-4, GAG-POL* were associated with B-induced alleviation of Al-toxicity.	[115]
*Pisum sativum* L. cv Zhongwan	50 µM Al, 24 h	50 µM B, 2 days	Increased chlorophyll and biomass; reduced chlorosis; reduced Al concentration in shoots; inhibited Al-binding in cell wall; reduced toxicity effect in roots and shoots.	[114]
*Vigna unguicula* L.	10 mM Al, 16 days	2.50 mM Si, 26 days	Increased *SOD, CAT, APX* and *POX* activities; Reduced Al contents of all tissues; Mitigated toxic effects of Al.	[134]
*Oriza sativa* L.	50 μM Al, 7 days	10 μM Si, 7 days	Restricted the uptake and transport of toxic Al in roots and leaves; maintained Mg and Zn at optimum levels; reduced cellular injury from Al toxicity.	[135]

## Abbreviations

Al	Aluminum
*ALMT1*	Aluminum-activated malate transporter 1
*AtMGT1*	*Arabidopsis* magnesium transport gene 1
BC	Base cation
Ca	Calcium
PG	Phosphogypsum
G	Gypsum
Mg	Magnesium
P	Phosphorus
S	Sulfur
B	Boron
Mg	Manganese
Zn	Zinc
Mo	Molybdenum
Si	Silicon
OAs	Organic acids
IAA	Indole acetic acid
PA	Polyamine
SA	Salicylic acid
Put	Putrescine
MF	Micorrhizal fungi
PGPB	Plant growth promoting bacteria

## References

[1] Kochian L.V., Piñeros M.A., Liu J., Magalhaes J.V. (2015). Plant adaptation to aid soils: The molecular basis for crop aluminum resistance. Annu. Rev. Plant Biol..

[2] Von Uexküll H.R., Mutert E., Date R.A., Grundon N.J., Rayment G.E., Probert M.E. (1995). Global extent, development and economic impact of acid soils. Plant-Soil Interactions at Low pH: Principles and Management, Proceedings of the Third International Symposium on Plant-Soil Interactions at Low pH, Brisbane, Queensland, Australia, 12–16 September 1993.

[3] Bojórquez-Quintal E., Escalante-Magaña C., Echevarría-Machado I., Martínez-Estévez M. (2017). Aluminum, a friend or foe of higher plants in acid soils. Front. Plant Sci..

[4] Dashuan T., Shuli N. (2015). A global analysis of soil acidification caused by nitrogen addition. Environ. Res. Lett..

[5] Goulding K.W.T. (2016). Soil acidification and the importance of liming agricultural soils with particular reference to the United Kingdom. Soil Use Manag..

[6] Singh S., Tripathi D.K., Singh S., Sharma S., Dubey N.K., Chauhan D.K., Vaculík M. (2017). Toxicity of aluminium on various levels of plant cells and organism: A review. Environ. Exp. Bot..

[7] Mossor-Pietraszewska T. (2001). Effect of aluminium on plant growth and metabolism. Acta Biochim. Pol..

[8] Kisnieriené V., Lapeikaité I. (2015). When chemistry meets biology: The case of aluminium—A review. Chemija.

[9] Grevenstuk T., Romano A. (2013). Aluminium speciation and internal detoxification mechanisms in plants: Where do we stand?. Metallomics.

[10] Frankowski M. (2016). Aluminum uptake and migration from the soil compartment into *Betula pendula* for two different environments: A polluted and environmentally protected area of Poland. Environ. Sci. Pollut. Res. Int..

[11] Clarkson D.T. (1965). The Effect of aluminium and some other trivalent metal cations on cell division in the root apices of *Allium Cepa*. Ann. Bot..

[12] Mariano E.D., Keltjens W.G. (2005). Long-term effects of aluminum exposure on nutrient uptake by maize genotypes differing in aluminum resistance. J. Plant Nutr..

[13] Chen Z.C., Liao H. (2016). Organic acid anions: An effective defensive weapon for plants against aluminum toxicity and phosphorus deficiency in acidic soils. J. Genet. Genom..

[14] Matsumoto H., Motoda H. (2012). Aluminum toxicity recovery processes in root apices. Possible association with oxidative stress. Plant Sci..

[15] Horst W.J., Asher C.J., Cakmak I., Szulkiewicz P., Wissemeier A.H. (1992). Short-term responses of soybean roots to aluminium. J. Plant Physiol..

[16] Kuo M.C., Kao C.H. (2003). Aluminum effects on lipid peroxidation and antioxidative enzyme activities in rice leaves. Biol. Plant..

[17] Devi S.R., Yamamoto Y., Matsumoto H. (2003). An intracellular mechanism of aluminum tolerance associated with high antioxidant status in cultured tobacco cells. J. Inorg. Biochem..

[18] Rengel Z. (2004). Aluminium cycling in the soil-plant-animal-human continuum. Biometals.

[19] Sivaguru M., Fujiwara T., Šamaj J., Baluška F., Yang Z., Osawa H., Maeda T., Mori T., Volkmann D., Matsumoto H. (2000). Aluminum-induced 1→3-β-d-glucan Inhibits cell-to-cell trafficking of molecules through plasmodesmata. A new mechanism of aluminum toxicity in plants. Plant Physiol..

[20] Yamamoto Y., Kobayashi Y., Devi S.R., Rikiishi S., Matsumoto H. (2002). Aluminum toxicity is associated with mitochondrial dysfunction and the production of reactive oxygen species in plant cells. Plant Physiol..

[21] Yang L.-T., Qi Y.-P., Jiang H.-X., Chen L.-S. (2013). Roles of organic acid anion secretion in aluminium tolerance of higher plants. BioMed Res. Int..

[22] Klug B., Specht A., Horst W.J. (2011). Aluminium localization in root tips of the aluminium-accumulating plant species buckwheat (*Fagopyrum esculentum* Moench). J. Exp. Bot..

[23] Wang S., Ren X., Huang B., Wang G., Zhou P., An Y. (2016). Aluminium-induced reduction of plant growth in alfalfa (*Medicago sativa*) is mediated by interrupting auxin transport and accumulation in roots. Sci. Rep..

[24] Klug B., Horst Walter J. (2010). Oxalate exudation into the root-tip water free space confers protection from aluminum toxicity and allows aluminum accumulation in the symplast in buckwheat (*Fagopyrum esculentum*). New Phytol..

[25] Arunakumara K.K.I.U., Walpola B.C., Yoon M.-H. (2013). Aluminum toxicity and tolerance mechanism in cereals and legumes—A review. J. Korean Soc. Appl. Biol. Chem..

[26] Xu J.M., Fan W., Jin J.F., Lou H.Q., Chen W.W., Yang J.L., Zheng S.J. (2017). Transcriptome analysis of al-induced genes in buckwheat (*Fagopyrum esculentum* Moench) root apex: New insight into al toxicity and resistance mechanisms in an al accumulating species. Front. Plant Sci..

[27] Nunes-Nesi A., Brito D.S., Inostroza-Blancheteau C., Fernie A.R., Araújo W.L. (2014). The complex role of mitochondrial metabolism in plant aluminum resistance. Trends Plant Sci..

[28] Ma J.F., Ryan P.R., Delhaize E. (2001). Aluminium tolerance in plants and the complexing role of organic acids. Trends Plant Sci..

[29] Lazof D.B., Goldsmith J.G., Rufty T.W., Linton R.W. (1994). Rapid uptake of aluminum into cells of intact soybean root tips (a microanalytical study using secondary ion mass spectrometry). Plant Physiol..

[30] Mora M.L., Alfaro M.A., Jarvis S.C., Demanet R., Cartes P. (2006). Soil aluminium availability in Andisols of southern Chile and its effect on forage production and animal metabolism. Soil Use Manag..

[31] Chaney R.I., Yosef B., Barrow N.J., Goldshmid J. (1989). Toxic element accumulation in soil and crop, protecting soil fertility and agricultural food-chains. Inorganics Contaminants in the Vadose Zone.

[32] Wang J.-P., Raman H., Zhang G.-P., Mendham N., Zhou M.-X. (2006). Aluminium tolerance in barley (*Hordeum vulgare* L.): Physiological mechanisms, genetics and screening methods. J. Zhejiang Univ. Sci. B.

[33] Matsumoto H., Hirasawa E., Morimura S., Takahashi E. (1976). Localization of aluminium in tea leaves. Plant Cell Physiol..

[34] Mehra A., Baker C.L. (2007). Leaching and bioavailability of aluminium, copper and manganese from tea (*Camellia sinensis*). Food Chem..

[35] Wills S.J., Sigel H., Sigel A. (1998). Aluminum toxicity and chronic renal failure. Metal Ions in Biological Systems. Aluminium and Its Role in Biology.

[36] Stewart W., Massey R.C., Taylor D. (1989). Aluminium toxicity in individuals with chronic renal disease. Aluminium in Food and the Environment.

[37] Anitha S., Rao K.S.J., Roesky H.W., Atwood D.A. (2002). The complexity of aluminum-DNA interactions: Relevance to Alzheimer’s and other neurological diseases. Group 13 Chemistry II: Biological Aspects of Aluminum.

[38] Kawahara M., Konoha K., Nagata T., Sadakane Y. (2007). Aluminum and human health: Its intake, bioavailability and neurotoxicity. Biomed. Res. Trace Elem..

[39] Klotz K., Weistenhöfer W., Neff F., Hartwig A., van Thriel C., Drexler H. (2017). The health effects of aluminum exposure. Dtsch. Arztebl. Int..

[40] Collard B.C.Y., Mackill D.J. (2008). Marker-assisted selection: An approach for precision plant breeding in the twenty-first century. Philos. Trans. R. Soc. Lond. B Biol. Sci..

[41] Vance C.P., Uhde-Stone C., Allan D.L. (2003). Phosphorus acquisition and use: Critical adaptations by plants for securing a nonrenewable resource. New Phytol..

[42] Gupta N., Gaurav S.S., Kumar A. (2013). Molecular basis of aluminium toxicity in plants: A review. Am. J. Plant Sci..

[43] Rahman M.A., Chikushi J., Duxbury J.M., Meisner C.A., Lauen J.G., Yasunaga E. (2005). Chemical control of soil environment by lime and nutrients to improve the productivity of acidic alluvial soils under rice-wheat cropping system in Bangladesh. Environ. Cont. Biol..

[44] Akhtaruzzaman M., Haque M.E., Osman K.T. (2014). Morphological, physical and chemical characteristics of hill forest soils at Chittagong University, Bangladesh. Open J. Soil Sci..

[45] Flues M., Sato I.M., Cotrim M.B., Salvador V.L., Ranzani A.C., Vallilo M.I., de Oliveira E. (2004). Soil characterization in a subtropical forest crossed by highways (Cantareira State Park, SP, Brazil). J. Braz. Chem. Soc..

[46] Ministry of Environmental Protection of the People’s Republic of China (MEPPRC) (1995). Environmental Quality Standard for Soils (GB 15618-1995).

[47] Wang S., Wang P., Fan C.Q. (2015). Distribution of aluminum fractionation in the acidic rhizosphere soils of masson pine (*Pinus massoniana* Lamb.). Commun. Soil Sci. Plant Anal..

[48] Xie Z., Chen Z., Sun W., Guo X., Yin B., Wang J. (2007). Distribution of aluminum and fluoride in tea plant and soil of tea garden in central and southwest China. Chin. Geogr. Sci..

[49] Soon Y. (1995). Forms of extractable aluminium in Canadian acid soils and their relations to plant growth. Plant-Soil Interactions at Low pH: Principles and Management.

[50] Bradová M., Tejnecký V., Borůvka L., Němeček K., Ash C., Šebek O., Svoboda M., Zenáhlíková J., Drábek O. (2015). The variations of aluminium species in mountainous forest soils and its implications to soil acidification. Environ. Sci. Pollut. Res. Int..

[51] Fichter J., Turpault M.-P., Dambrine E., Ranger J. (1998). Mineral evolution of acid forest soils in the Strengbach catchment (Vosges mountains, N-E France). Geoderma.

[52] Bera R., Seal A., Banerjee M., Dolui A.K. (2005). Nature and profile distribution of iron and aluminum in relation to pedogenic processes in some soils developed under tropical environment in India. Environ. Geol..

[53] McGrath D., Fleming G.A., Culleton N. (2008). Trace Elements and Heavy Metals in Irish Soils.

[54] Yanai J., Okada T., Yamada H. (2012). Elemental composition of agricultural soils in Japan in relation to soil type, land use and region. Soil Sci. Plant Nutr..

[55] Kim J.G., Lee S.S., Moon H.-S., Kang I.M. (2002). Land application of alum sludge from water purification plant to acid mineral soil treated with acidic water. Soil Sci. Plant Nutr..

[56] Lee C.H., Lee S.-W., Kim E.-Y., Jeong J.-H., Cho H.-J., Park G.-S., Lee C.-Y., Jeong Y.-H. (2005). Effects of air pollution and acid deposition on three *Pinus densiflora* (Japanese red pine) forests in South Korea. J. Agric. Meteorol..

[57] Moir J., Moot D. (2014). Medium-term soil pH and exchangeable aluminium response to liming at three high country locations. Proceedings of the New Zealand Grassland Association Conference.

[58] Brown T.T., Koenig R.T., Huggins D.R., Harsh J.B., Rossi R.E. (2008). Lime effects on soil acidity, crop yield, and aluminum chemistry in direct-seeded cropping systems. Soil Sci. Soc. Am. J..

[59] Lee Y.-B., Bigham J.M., Kim P.-J. (2007). Evaluate changes in soil chemical properties following FGD-gypsum application. Korean J. Environ. Agric..

[60] Jaiswal S.K., Naamala J., Dakora F.D. (2018). Nature and mechanisms of aluminium toxicity, tolerance and amelioration in symbiotic legumes and rhizobia. Biol. Fert. Soils.

[61] Vitorello V.A., Capaldi F.R., Stefanuto V.A. (2005). Recent advances in aluminum toxicity and resistance in higher plants. Braz. J. Plant Physiol..

[62] Delhaize E., Ryan P.R. (1995). Aluminum toxicity and tolerance in plants. Plant Physiol..

[63] Brautigan D.J., Rengasamy P., Chittleborough D.J. (2012). Aluminium speciation and phytotoxicity in alkaline soils. Plant Soil.

[64] Kinraide T.B. (1990). Assessing the rhizotoxicity of the aluminate Ion,Al(OH)_4_. Plant Physiol..

[65] Rengel Z. (1996). Uptake of aluminum by plant cells. New Phytol..

[66] Kopittke P.M., Moore K.L., Lombi E., Gianoncelli A., Ferguson B.J., Blamey F.P.C., Menzies N.W., Nicholson T.M., McKenna B.A., Wang P. (2015). Identification of the primary lesion of toxic aluminum in plant roots. Plant Physiol..

[67] Ma J.F., Hiradate S. (2000). Form of aluminium for uptake and translocation in buckwheat (*Fagopyrum esculentum* Moench). Planta.

[68] Wang Y., Li R., Li D., Jia X., Zhou D., Li J., Lyi S.M., Hou S., Huang Y., Kochian L.V. (2017). *NIP1;2* is a plasma membrane-localized transporter mediating aluminum uptake, translocation, and tolerance in *Arabidopsis*. Proc. Natl. Acad. Sci. USA.

[69] Chenery E.M. (1948). Aluminium in the Plant World. Kew Bull..

[70] Chenery E.M. (1949). Aluminium in the Plant World. Kew Bull..

[71] Chen R.F., Shen R.F., Gu P., Wang H.Y., Xu X.H. (2008). Investigation of aluminum-tolerant species in acid soils of South China. Commun. Soil Sci. Plant Anal..

[72] Ownby J.D., Popham H.R. (1989). Citrate reverses the inhibition of wheat root growth caused by aluminum. J. Plant Physiol..

[73] Palmer A.J., Baker A., Muench S.P. (2016). The varied functions of aluminium-activated malate transporters-much more than aluminium resistance. Biochem. Soc. Trans..

[74] Yokosho K., Yamaji N., Fujii-Kashino M., Ma J.F. (2016). Retrotransposon-mediated aluminum tolerance through enhanced expression of the citrate transporter *OsFRDL4*. Plant Physiol..

[75] Wu X., Li R., Shi J., Wang J., Sun Q., Zhang H., Xing Y., Qi Y., Zhang N., Guo Y.-D. (2014). *Brassica oleracea* MATE Encodes a citrate transporter and enhances aluminum tolerance in *Arab. Thaliana*. Plant Cell Physiol..

[76] Kang D.-J., Seo Y.-J., Futakuchi K., Vijarnsorn P., Ishii R. (2011). Effect of aluminum toxicity on flowering time and grain yield on rice genotypes differing in Al-tolerance. J. Crop. Sci. Biotechnol..

[77] Tan K., Keltjens W.G., Findenegg G.R. (1993). Aluminum toxicity in sorghum genotypes as influenced by solution acidity. Soil Sci. Plant Nutr..

[78] Silva S., Pinto-Carnide O., Martins-Lopes P., Matos M., Guedes-Pinto H., Santos C. (2010). Differential aluminium changes on nutrient accumulation and root differentiation in an Al sensitive vs. tolerant wheat. Environ. Exp. Bot..

[79] Islam M.A., Dowling P.M., Milham P.J., Campbell L.C., Jacobs B.C., Garden D.L. (2006). Ranking acidity tolerance and growth potential of Austrodanthonia accessions. Grassl. Sci..

[80] Pereira J.F. (2018). Initial root length in wheat is highly correlated with acid soil tolerance in the field. Sci. Agric..

[81] Koenig R., Schroeder K., Carter A., Pumphrey M., Paulitz T., Campbell K., Huggins D. (2011). Soil Acidity and Aluminum Toxicity in the Palouse Region of the Pacific Northwest.

[82] Leidi E., Rodrıguez-Navarro D.N., Fernández M., Sarmiento R., Semedo J., Marques N., Matos A., Machado A.P., Ørting B., Sørensen M. (2004). Factors affecting root and seed yield in ahipa (*Pachyrhizus ahipa* (Wedd.) Parodi), a multipurpose legume crop. Eur. J. Agron..

[83] Ishikawa S., Wagatsuma T. (1998). Plasma membrane permeability of root-tip cells following temporary exposure to al ions is a rapid measure of al tolerance among plant species. Plant Cell Physiol..

[84] Nagy N.E., Dalsen L.S., Jones D.L., Swensen B., Fossdal C.G., Eldhuset T.D. (2004). Cytological and enzymatic responses to aluminium stress in root tips of Norway spruce seedlings. New Phytol..

[85] Watanabe T., Osaki M. (2002). Role of organic acids in aluminum accumulation and plant growth in *Melastoma Malabathricum*. Tree Physiol..

[86] Kaneko M., Yoshimura E., Nishizawa N.K., Mori S. (1999). Time course study of aluminum-induced callose formation in barley roots as observed by digital microscopy and low-vacuum scanning electron microscopy. Soil Sci. Plant Nutr..

[87] Rangel A.F., Madhusudana R.I., Johannes H.W. (2009). Intracellular distribution and binding state of aluminum in root apices of two common bean (*Phaseolus vulgaris*) genotypes in relation to Al toxicity. Physiol. Plant..

[88] McLean F.T., Gilbert B.E. (1927). The relative aluminum tolerance of crop plants. Soil Sci..

[89] Parrot W., Bouton J. (1990). Aluminum tolerance in alfalfa as expressed in tissue culture. Crop Sci..

[90] Khatiwada S.P., Senadhira D., Carpena A.L., Zeigler R.S., Fernandez P.G. (1996). Variability and genetics of tolerance for aluminum toxicity in rice (*Oryza sativa* L.). Theor. Appl. Genet..

[91] Garvin D.F., Carver B.F. (2003). Role of the genotype in tolerance to acidity and aluminum toxicity. Handbook of Soil Acidity.

[92] Famoso A.N., Clark R.T., Shaff J.E., Craft E., McCouch S.R., Kochian L.V. (2010). Development of a novel aluminum tolerance phenotyping platform used for comparisons of cereal aluminum tolerance and investigations into rice aluminum tolerance mechanisms. Plant Physiol..

[93] Taylor G.J., Blarney F.P.C., Edwards D.G. (1998). Antagonistic and synergistic interactions between aluminum and manganese on growth of *Vigna unguiculata* at low ionic strength. Physiol. Plant..

[94] Bolan N.S., Hedley M.J., White R.E. (1991). Processes of soil acidification during nitrogen cycling with emphasis on legume based pastures. Plant Soil.

[95] Veitch F.P. (1904). Comparison of methods for the estimation of soil acidity. J. Am. Chem. Soc..

[96] Driscoll C.T., Driscoll K.M., Mitchell M.J., Raynal D.J. (2003). Effects of acidic deposition on forest and aquatic ecosystems in New York State. Environ. Pollut..

[97] Brunner I., Sperisen C. (2013). Aluminum exclusion and aluminum tolerance in woody plants. Front. Plant Sci..

[98] Nawaz R., Parkpian P., Garivait H., Anurakpongsatorn P., DeLaune R.D., Jugsujinda A. (2012). Impacts of acid rain on base cations, aluminum, and acidity development in highly weathered soils of Thailand. Commun. Soil Sci. Plant Anal..

[99] Rengel Z., Rengel Z. (2002). Role of pH in Availability of Ions in Soil. Handbook of Plant Growth pH as the Master Variable.

[100] Stass A., Wang Y., Eticha D., Horst W.J. (2006). Aluminium rhizotoxicity in maize grown in solutions with Al^3+^ or Al(OH)_4_− as predominant solution Al species. J. Exp. Bot..

[101] Horst W.J., Püschel A.-K., Schmohl N. (1997). Induction of callose formation is a sensitive marker for genotypic aluminium sensitivity in maize. Plant Soil.

[102] Gensemer R.W., Playle R.C. (1999). The bioavailability and toxicity of aluminum in aquatic environments. Crit. Rev. Environ. Sci. Technol..

[103] Driscoll C.T., Schecher W.D. (1990). The chemistry of aluminum in the environment. Environ. Geochem. Health.

[104] Driscoll C.T., Schecher W.D., Gitelman H.J. (1989). Aqueous Chemistry of Aluminium. Aluminium and Health: A Critical Review.

[105] Adams M.L. (1999). Speciation and Measurement of Aluminium in Environmental Systems. Ph.D. Thesis.

[106] Olivares E., Peña E., Marcano E., Mostacero J., Aguiar G., Benítez M., Rengifo E. (2009). Aluminum accumulation and its relationship with mineral plant nutrients in 12 pteridophytes from Venezuela. Environ. Exp. Bot..

[107] De Mendonça R.J., Cambraia J., de Oliveira J.A., Oliva M.A. (2003). Efeito do alumínio na absorção e na utilização de macronutrientes em duas cultivares de arroz. Pesqui. Agropecu. Bras..

[108] Simon L., Smalley T.J., Jones J.B., Lasseigne F.T. (1994). Aluminum toxicity in tomato. Part 1. Growth and mineral nutrition. J. Plant Nutr..

[109] Zobel R.W., Kinraide T.B., Baligar V.C. (2007). Fine root diameters can change in response to changes in nutrient concentrations. Plant Soil.

[110] Poschenrieder C., Llugany M., Barceló J. (1995). Short-term effects of pH and aluminium on mineral nutrition in maize varieties differing in proton and aluminium tolerance. J. Plant Nutr..

[111] Brady D.J., Edwards D.G., Asher C.J., Blamey F.P.C. (1993). Calcium amelioration of aluminium toxicity effects on root hair development in soybean [*Glycine max* (L.) Merr.]. New Phytol..

[112] Edmeades D., Wheeler D., Blamey F., Christie R. (1991). Calcium and Magnesium Amelioration of Aluminium Toxicity in Al-sensitive and Al-tolerant Wheat. Plant-Soil Interactions at Low pH.

[113] Silva I.R., Smyth T.J., Israel D.W., Raper C.D., Rufty T.W. (2001). Magnesium ameliorates aluminum rhizotoxicity in soybean by increasing citric acid production and exudation by roots. Plant Cell Physiol..

[114] Yu M., Shen R., Xiao H., Xu M., Wang H., Wang H., Zeng Q., Bian J. (2009). Boron alleviates aluminum toxicity in pea (*Pisum sativum*). Plant Soil.

[115] Zhou X.-X., Yang L.-T., Qi Y.-P., Guo P., Chen L.-S. (2015). Mechanisms on boron-induced alleviation of aluminum-toxicity in citrus grandis seedlings at a transcriptional level revealed by cDNA-AFLP analysis. PLoS ONE.

[116] Guo P., Li Q., Qi Y.-P., Yang L.-T., Ye X., Chen H.-H., Chen L.-S. (2017). Sulfur-mediated-alleviation of aluminum-toxicity in *Citrus grandis* seedlings. Int. J. Mol. Sci..

[117] Teng W., Kang Y., Hou W., Hu H., Luo W., Wei J., Wang L., Zhang B. (2018). Phosphorus application reduces aluminum toxicity in two *Eucalyptus* clones by increasing its accumulation in roots and decreasing its content in leaves. PLoS ONE.

[118] Panhwar Q.A., Naher U.A., Radziah O., Shamshuddin J., Razi I.M. (2014). Bio-fertilizer, ground magnesium limestone and basalt applications may improve chemical properties of Malaysian acid sulfate soils and rice growth. Pedosphere.

[119] Panhwar Q.A., Naher U.A., Radziah O., Shamshuddin J., Razi I.M. (2015). Eliminating aluminum toxicity in an acid sulfate soil for rice cultivation using plant growth promoting bacteria. Molecules.

[120] Noble A.D., Sumner M.E. (1988). Calcium and Al interactions and soybean growth in nutrient solutions. Commun. Soil Sci. Plant Anal..

[121] Kinraide T.B. (1998). Three mechanisms for the calcium alleviation of mineral toxicities. Plant Physiol..

[122] Hossain M.A., Ashrafuzzaman M., Hossain A.K.M.Z., Ismail M.R., Koyama H. (2014). Role of accumulated calcium in alleviating aluminum injury in wheat plants. Sci. World J..

[123] Vicensi M., Müller M.M.L., Kawakami J., de Nascimento R., Michalovicz L., Lopes C. (2016). Do rates and splitting of phosphogypsum applications influence the soil and annual crops in a no-tillage system?. Rev. Bras. Ciênc. Solo.

[124] Alva A.K., Sumner M.E. (1990). Amelioration of acid soil infertility by phosphogypsum. Plant Soil.

[125] Pavan M.A., Bingham F.T., Peryea F.J. (1987). Influence of calcium and magnesium salts on acid soil chemistry and calcium nutrition of Apple. Soil Sci. Soc. Am. J..

[126] Smith C., Peoples M., Keerthisinghe G., James T., Garden D., Tuomi S. (1994). Effect of surface applications of lime, gypsum and phosphogypsum on the alleviating of surface and subsurface acidity in a soil under pasture. Soil Res..

[127] Carvalho M.C.S., van Raij B. (1997). Calcium sulphate, phosphogypsum and calcium carbonate in the amelioration of acid subsoils for root growth. Plant Soil.

[128] Mora M.L., Schnettler B., Demanet R. (1999). Effect of liming and gypsum on soil chemistry, yield, and mineral composition of ryegrass grown in an acidic Andisol. Commun. Soil Sci. Plant Anal..

[129] Reyes-Díaz M., Meriño-Gergichevich C., Alarcón E., Alberdi M., Horst W.J. (2011). Calcium sulfate ameliorates the effect of aluminum toxicity differentially in genotypes of highbush blueberry (*Vaccinium corymbosum* L.). J. Soil Sci. Plant Nutr..

[130] Syed-Omar S.R., Shamsuddin Z.H., Zuraidah J.Y., Wynne J.C., Elkan G.H., Wright R.J., Baligar V.C., Murrmann R.P. (1991). Use of Lime, Gypsum and Their Combinations to Improve Iodulation and Yield of Groundnut in an Acidic Soil. Plant-Soil Interactions at Low pH, Proceedings of the Second International Symposium on Plant-Soil Interactions at Low pH, Beckley, VA, USA, 24–29 June 1990.

[131] Tirado-Corbalá R., Slater B.K., Dick W.A., Barker D. (2017). Alfalfa responses to gypsum application measured using undisturbed soil columns. Plants.

[132] Farina M., Channon P. (1988). Acid-subsoil amelioration: II. Gypsum effects on growth and subsoil chemical properties. Soil Sci. Soc. Am. J..

[133] Kinraide T.B., Pedler J.F., Parker D.R. (2004). Relative effectiveness of calcium and magnesium in the alleviation of rhizotoxicity in wheat induced by copper, zinc, aluminum, sodium, and low pH. Plant Soil.

[134] De Jesus L.R., Batista B.L., da Silva Lobato A.K. (2017). Silicon reduces aluminum accumulation and mitigates toxic effects in cowpea plants. Acta Physiol. Plant..

[135] Singh V.P., Tripathi D.K., Kumar D., Chauhan D.K. (2011). Influence of exogenous silicon addition on aluminium tolerance in rice seedlings. Biol. Trace Elem. Res..

[136] Manson A., Findlay N. (2015). Agricultural Uses of Lime and Gypsum.

[137] Mora M.L., Cartes P., Demanet R., Cornforth I.S. (2002). Effects of lime and gypsum on pasture growth and composition on an acid Andisol in Chile, South America. Commun. Soil Sci. Plant Anal..

[138] Rashad A.M. (2017). Phosphogypsum as a construction material. J. Clean. Prod..

[139] Campbell C.G., Garrido F., Illera V., García-González M.T. (2006). Transport of Cd, Cu and Pb in an acid soil amended with phosphogypsum, sugar foam and phosphoric rock. Appl. Geochem..

[140] Toma M., Saigusa M. (1997). Effects of phosphogypsum on amelioration of strongly acid nonallophanic Andosols. Plant Soil.

[141] Garrido F., Illera V., Vizcayno C., García-González M.T. (2003). Evaluation of industrial by-products as soil acidity amendments: Chemical and mineralogical implications. Eur. J. Soil Sci..

[142] Mays D.A., Mortvedt J.J. (1986). Crop response to soil applications of phosphogypsum. J. Environ. Qual..

[143] Korcak R.F. (1988). Fluidized bed material applied at disposal levels: Effects on an apple orchard. J. Environ. Qual..

[144] Tuna A.L., Kaya C., Ashraf M., Altunlu H., Yokas I., Yagmur B. (2007). The effects of calcium sulphate on growth, membrane stability and nutrient uptake of tomato plants grown under salt stress. Environ. Exp. Bot..

[145] Toma M., Saigusa M., Qafoku N., Sumner M. (2005). Effects of gypsum on amelioration of subsoil acidity in Andisols. J. Integr. Field Sci..

[146] Shamshuddin J., Fauziah I.C., Sharifuddin H.A.H. (1991). Effects of limestone and gypsum application to a Malaysian ultisol on soil solution composition and yields of maize and groundnut. Plant Soil.

[147] Sanderson K.R., Eaton L.J. (2004). Gypsum-an alternative to chemical fertilizers in lowbush blueberry production. Small Fruits Rev..

[148] Korcak R.F. (1993). Short-term response of blueberry to elevated soil calcium. J. Small Fruit Vitic..

[149] Rengel Z., Bose J., Chen Q., Tripathi B.N. (2015). Magnesium alleviates plant toxicity of aluminium and heavy metals. Crop. Pasture Sci..

[150] Bose J., Babourina O., Rengel Z. (2011). Role of magnesium in alleviation of aluminium toxicity in plants. J. Exp. Bot..

[151] Grauer U.E., Horst W.J. (1992). Modeling cation amelioration of aluminum phytotoxicity. Soil Sci. Soc. Am. J..

[152] Bose J., Babourina O., Shabala S., Rengel Z. (2013). Low-pH and aluminum resistance in Arabidopsis correlates with high cytosolic magnesium content and increased magnesium uptake by plant roots. Plant Cell Physiol..

[153] Palmgren M.G. (2001). Plant plasmamembrane *H+-ATPases*: Powerhouses for nutrient uptake. Annu. Rev. Plant Phys..

[154] Kinoshita T., Shimazaki K.I. (1999). Blue light activates the plasma membrane *H^(+)^-ATPase* by phosphorylation of the C-terminus in stomatal guard cells. EMBO J..

[155] Rober-Kleber N., Albrechtová J.T.P., Fleig S., Huck N., Michalke W., Wagner E., Speth V., Neuhaus G., Fischer-Iglesias C. (2003). Plasma membrane *H^+^-ATPase* is involved in auxin-mediated cell elongation during wheat embryo development. Plant Physiol..

[156] Yang J.L., You J.F., Li Y.Y., Wu P., Zheng S.J. (2007). Magnesium enhances aluminum-induced citrate secretion in rice bean roots (*Vigna umbellata*) by restoring plasma membrane *H^+^-ATPase* activity. Plant Cell Physiol..

[157] Chen Q., Kan Q., Wang P., Yu W., Yu Y., Zhao Y., Yu Y., Li K., Chen L. (2015). Phosphorylation and interaction with the *14-3-3* protein of the plasma membrane *H^+^-ATPase* are involved in the regulation of magnesium-mediated increases in aluminum-induced citrate exudation in broad bean (*Vicia faba*. L). Plant Cell Physiol..

[158] Kellermayer R., Aiello D.P., Miseta A., Bedwell D.M. (2003). Extracellular Ca^2+^ sensing contributes to excess Ca^2+^ accumulation and vacuolar fragmentation in a *PMR1Δ* mutant of *S*. *Cerevisiae*. J. Cell Sci..

[159] Brüggemann L.I., Pottosin I.I., Schönknecht G. (1999). Cytoplasmic magnesium regulates the fast activating vacuolar cation channel. J. Exp. Bot..

[160] Deng W., Luo K., Li D., Zheng X., Wei X., Smith W., Thammina C., Lu L., Li Y., Pei Y. (2006). Overexpression of an *Arabidopsis* magnesium transport gene, *AtMGT1*, in *Nicotiana benthamiana* confers Al tolerance. J. Exp. Bot..

[161] Razaq M., Zhang P., Shen H.-L., Salahuddin (2017). Influence of nitrogen and phosphorous on the growth and root morphology of Acer mono. PLoS ONE.

[162] Tariq A., Pan K., Olatunji O.A., Graciano C., Li Z., Sun F., Sun X., Song D., Chen W., Zhang A. (2017). Phosphorous application improves drought tolerance of *Phoebe Zhennan*. Front. Plant Sci..

[163] Tan K., Keltjens W.G. (1990). Interaction between aluminium and phosphorus in sorghum plants. Plant Soil.

[164] Zheng S.J., Yang J.L., He Y.F., Yu X.H., Zhang L., You J.F., Shen R.F., Matsumoto H. (2005). Immobilization of aluminum with phosphorus in roots is associated with high aluminum resistance in buckwheat. Plant Physiol..

[165] Iqbal M.T. (2013). Phosphorus enhances aluminium tolerance in both aluminium-tolerant and aluminium-sensitive wheat seedlings. S. Afr. J. Plant Soil.

[166] Chen R.F., Zhang F.L., Zhang Q.M., Sun Q.B., Dong X.Y., Shen R.F. (2012). Aluminium-phosphorus interactions in plants growing on acid soils: Does phosphorus always alleviate aluminium toxicity?. J. Sci. Food Agric..

[167] Ch’ng H.Y., Ahmed O.H., Majid N.M.A. (2014). Improving phosphorus availability in an acid soil using organic amendments produced from agroindustrial wastes. Sci. World J..

[168] Wang X., Yan X., Liao H. (2010). Genetic improvement for phosphorus efficiency in soybean: A radical approach. Ann. Bot..

[169] Scott N.M., Vaughan D., Malcolm R.E. (1985). Sulphur in Soils and Plants. Soil Organic Matter and Biological Activity.

[170] Dixit G., Singh A.P., Kumar A., Mishra S., Dwivedi S., Kumar S., Trivedi P.K., Pandey V., Tripathi R.D. (2016). Reduced arsenic accumulation in rice (*Oryza sativa* L.) shoot involves sulfur mediated improved thiol metabolism, antioxidant system and altered arsenic transporters. Plant Physiol. Biochem..

[171] Saifullah, Khan M.N., Iqbal M., Naeem A., Bibi S., Waraich E.A., Dahlawi S. (2016). Elemental sulfur improves growth and phytoremediative ability of wheat grown in lead-contaminated calcareous soil. Int. J. Phytoremediat..

[172] Zhang H., Tan Z.-Q., Hu L.-Y., Wang S.-H., Luo J.-P., Jones R.L. (2010). Hydrogen sulfide alleviates aluminum toxicity in germinating wheat seedlings. J. Integr. Plant Biol..

[173] Dawood M., Cao F., Jahangir M.M., Zhang G., Wu F. (2012). Alleviation of aluminum toxicity by hydrogen sulfide is related to elevated *ATPase*, and suppressed aluminum uptake and oxidative stress in barley. J. Hazard. Mater..

[174] Qian P., Sun R., Ali B., Gill R.A., Xu L., Zhou W. (2014). Effects of hydrogen sulfide on growth, antioxidative capacity, and ultrastructural changes in oilseed rape seedlings under aluminum toxicity. J. Plant Growth Regul..

[175] Zhang J., Zhao C.-Y., Liu J., Song R., Du Y.-X., Li J.-Z., Sun H.-Z., Duan G.-L., Zhao Q.-Z. (2016). Influence of sulfur on transcription of genes involved in arsenic accumulation in rice grains. Plant Mol. Biol. Rep..

[176] Skwierawska M., Zawartka L., Zawadzki B. (2008). The effect of different rates and forms of sulphur applied on changes of soil agrochemical properties. Plant Soil Environ..

[177] Hossain A.K.M.Z., Hossain M.A., Koyama H., Hara T. (2004). Effects of aluminum and boron supply on growth of seedlings among 15 cultivars of wheat (*Triticum aestivum* L.) grown in Bangladesh. Soil Sci. Plant Nutr..

[178] Riaz M., Wu X., Yan L., Hussain S., Aziz O., Shah A., Jiang C. (2018). Boron supply alleviates Al-induced inhibition of root elongation and physiological characteristics in rapeseed (*Brassica napus* L.). J. Plant Interact..

[179] Gupta U.C., Jame Y.W., Campbell C.A., Leyshon A.J., Nicholaichuk W. (1985). Boron toxicity and deficiency: A review. Can. J. Soil Sci..

[180] Epstein E. (1994). The anomaly of silicon in plant biology. Proc. Natl. Acad. Sci. USA.

[181] Farooq M.A., Dietz K.-J. (2015). Silicon as versatile player in plant and human biology: Overlooked and poorly understood. Front. Plant Sci..

[182] Farooq M.A., Saqib Z.A., Akhtar J., Bakhat H.F., Pasala R.-K., Dietz K.-J. (2015). Protective role of silicon (Si) against combined stress of salinity and boron (B) toxicity by improving antioxidant enzymes activity in rice. Silicon.

[183] Ma J.F. (2004). Role of silicon in enhancing the resistance of plants to biotic and abiotic stresses. Soil Sci. Plant Nutr..

[184] Qian L., Chen B., Chen M. (2016). Novel alleviation mechanisms of aluminum phytotoxicity via released biosilicon from rice straw-derived biochars. Sci. Rep..

[185] Kopittke P.M., Gianoncelli A., Kourousias G., Green K., McKenna B.A. (2017). Alleviation of Al toxicity by Si is associated with the formation of Al-Si complexes in root tissues of sorghum. Front. Plant Sci..

[186] Li J.-Y., Wang N., Xu R.-K., Tiwari D. (2010). Potential of industrial byproducts in ameliorating acidity and aluminum toxicity of soils under tea plantation. Pedosphere.

[187] An Y., Zhou P., Xiao Q., Shi D. (2014). Effects of foliar application of organic acids on alleviation of aluminum toxicity in alfalfa. J. Plant Nutr. Soil Sci..

[188] Wang Q., Nian F., Zhao L., Li F., Yang H., Yang Y. (2013). Exogenous indole-3-acetic acid could reduce the accumulation of aluminum in root apex of wheat (*Triticum aestivum* L.) under Al stress. J. Soil Sci. Plant Nutr..

[189] Wang S., Yuan S., Su L., Lv A., Zhou P., An Y. (2017). Aluminum toxicity in alfalfa (*Medicago sativa*) is alleviated by exogenous foliar IAA inducing reduction of Al accumulation in cell wall. Environ. Exp. Bot..

[190] Pandey P., Srivastava R.K., Dubey R.S. (2013). Salicylic acid alleviates aluminum toxicity in rice seedlings better than magnesium and calcium by reducing aluminum uptake, suppressing oxidative damage and increasing antioxidative defense. Ecotoxicology.

[191] Liu N., Song F., Zhu X., You J., Yang Z., Li X. (2017). Salicylic acid alleviates aluminum toxicity in soybean roots through modulation of reactive oxygen species metabolism. Front. Chem..

[192] Yu Y., Jin C., Sun C., Wang J., Ye Y., Zhou W., Lu L., Lin X. (2016). Inhibition of ethylene production by putrescine alleviates aluminium-induced root inhibition in wheat plants. Sci. Rep..

[193] Kochian L.V., Piñeros M.A., Hoekenga O.A. (2005). The physiology, genetics and molecular biology of plant aluminum resistance and toxicity. Plant Soil.

[194] Yang Z.-M., Wang J., Wang S.-H., Xu L.-L. (2003). Salicylic acid-induced aluminum tolerance by modulation of citrate efflux from roots of *Cassia tora* L.. Planta.

[195] Surapu V., Ediga A., Meriga B. (2014). Salicylic acid alleviates aluminum toxicity in tomato seedlings (*Lycopersicum esculentum* Mill.) through activation of antioxidant defense system and proline biosynthesis. Adv. Biosci. Biotechnol..

[196] Rouphael Y., Cardarelli M., Colla G. (2015). Role of arbuscular mycorrhizal fungi in alleviating the adverse effects of acidity and aluminium toxicity in zucchini squash. Sci. Hortic..

[197] Rufyikiri G., Declerck S., Dufey J.E., Delvaux B. (2000). Arbuscular mycorrhizal fungi might alleviate aluminium toxicity in banana plants. New Phytol..

